# De Novo Potent Peptide Nucleic Acid Antisense Oligomer Inhibitors Targeting SARS-CoV-2 RNA-Dependent RNA Polymerase via Structure-Guided Drug Design

**DOI:** 10.3390/ijms242417473

**Published:** 2023-12-14

**Authors:** Kiran Shehzadi, Mingjia Yu, Jianhua Liang

**Affiliations:** Key Laboratory of Medical Molecule Science and Pharmaceutical Engineering, Ministry of Industry and Information Technology, School of Chemistry and Chemical Engineering, Beijing Institute of Technology, Beijing 100811, China; kiranshehzadi@bit.edu.cn

**Keywords:** peptide nucleic acid, cell-penetrating peptide, RdRp, RdRp-RNA, SARS-CoV-2

## Abstract

Global reports of novel SARS-CoV-2 variants and recurrence cases continue despite substantial vaccination campaigns, raising severe concerns about COVID-19. While repurposed drugs offer some treatment options for COVID-19, notably, nucleoside inhibitors like Remdesivir stand out as curative therapies for COVID-19 that are approved by the US Food and Drug Administration (FDA). The emergence of highly contagious SARS-CoV-2 variants underscores the imperative for antiviral drugs adaptable to evolving viral mutations. RNA-dependent RNA polymerase (RdRp) plays a key role in viral genome replication. Currently, inhibiting viral RdRp function remains a pivotal strategy to tackle the notorious virus. Peptide nucleic acid (PNA) therapy shows promise by effectively targeting specific genome regions, reducing viral replication, and inhibiting infection. In our study, we designed PNA antisense oligomers conjugated with cell-penetrating peptides (CPP) aiming to evaluate their antiviral effects against RdRp target using structure-guided drug design, which involves molecular docking simulations, drug likeliness and pharmacokinetic evaluations, molecular dynamics simulations, and computing binding free energy. The in silico analysis predicts that chemically modified PNAs might act as antisense molecules in order to disrupt ribosome assembly at RdRp’s translation start site, and their chemically stable and neutral backbone might enhance sequence-specific RNA binding interaction. Notably, our findings demonstrate that PNA-peptide conjugates might be the most promising inhibitors of SARS-CoV-2 RdRp, with superior binding free energy compared to Remdesivir in the current COVID-19 medication. Specifically, PNA-CPP-1 could bind simultaneously to the active site residues of RdRp protein and sequence-specific RdRp-RNA target in order to control viral replication.

## 1. Introduction

**Figure 1:**
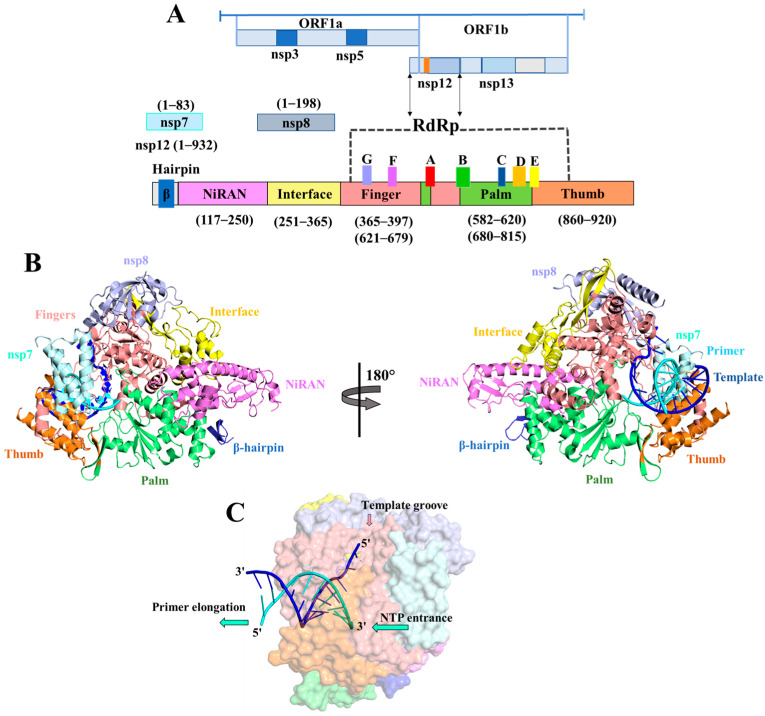
Structural architecture of SARS-CoV-2 RdRp domain. (**A**) SARS-CoV-2 genome structural organization: It encodes two large ORF1a and ORF1b genes, which encode non-structural proteins that form a replication–transcription complex. The non-structural protein 12 (nsp12) encodes RNA-dependent RNA-polymerase (RdRp). The schematic diagram for the components of the RdRp complex containing nsp12, nsp7, and nsp8. The polymerase motif (A to G) and the β hairpin specific to SARS-CoV-2 are colored red, green, blue, light orange, yellow, violet, light blue, and deep teal, respectively. (**B**) Two views of the RdRp-RNA cryo-EM structure (PDB ID: 7BV2) showing the arrangement of palm (green), fingers (light salmon), thumb (tv orange) domains along with nsp7 (cyan), nsp8 (grey blue), and NiRAN (violet) domain in cartoon model. (**C**) Surface view of RdRp-RNA protein complex, the entrance for nucleotide triphosphate (NTP), primer elongation direction, and template groove are presented.

The severe acute respiratory syndrome coronavirus 2 (SARS-CoV-2), a member of the *Betacoronavirus* family, has caused the rapid and extensive expansion of the ongoing coronavirus disease 2019 (COVID-19) pandemic [1]. The number of confirmed cases has surpassed an alarming 768,237,788 with a staggering death toll of 6,951,677 (as of 19 July 2023) [2,3]. Despite the administration of over 12 billion vaccine doses, a significant portion of the global population remains unvaccinated [4]. Furthermore, individuals who have received the recommended three vaccine doses or those who have previously been infected can still fall victim to the virus due to its rapid mutations [5]. SARS-CoV-2 primarily spreads through respiratory droplets and contact transmission, highlighting the crucial role of respiratory hygiene and proper personal protective measures. While extensive efforts have been dedicated to the development of antiviral therapeutics, the clinical stage of most studies has been prematurely discontinued due to various factors, including the limited metabolic stability, inherent toxicity, and substantial side effects associated with current medications. Recently, few drugs and vaccines, including Tocilizumab, Baricitinib, Paxlovid (Nirmatrelvir and Ritonavir), along with Remdesivir, received approval from the FDA for the treatment of COVID-19 (https://www.fda.gov/drugs/emergency-preparedness-drugs/coronavirus-covid-19-drugs) (accessed on 2 November 2023). The COVID-19 pandemic remains a high priority for the Center of Drug Evaluation and Research (CDER). Despite widespread vaccination efforts and the availability of some antiviral drugs, the virus’s mutational dynamics have led to breakthrough infections even in vaccinated or previously infected individuals [6,7]. The development of new drugs to combat this lethal virus is an expensive and time-consuming process. Urgent attention is required to address the scarcity of approved drugs and further advance therapeutic research [8,9]. Moreover, the development and availability of essential medical resources, such as medications, vaccines, diagnostics, and chemical reagents, are imperative in the battle against SARS-CoV-2. The viral genome harbors at least 14 open reading frames (ORFs), which play pivotal roles in viral replication and propagation. Among these, ORF1a and ORF1b encode two major replicase polyproteins (PP1a and PP1ab) that undergo proteolytic cleavage, resulting in the generation of sixteen non-structural proteins (nsps) [10]. The replication of viral RNA genomic material is meticulously regulated by an essential enzyme nsp12, which is known as RNA-dependent RNA polymerase (RdRp), encoded by the viral genome itself. The polymerase domain of SARS-CoV-2 RdRp is made up of three subdomains: a fingers subdomain with residues ranging from 365 to 397 aa and 621 to 679 aa; a palm subdomain with residues ranging from 582 to 620 aa and 680 to 815 aa; and a thumb sub-domain whose residues are 860–920 aa. Its N-terminal extension domain with residues ranges from 117 to 250 aa and is specific to nidoviruses that assume a nidovirus RdRp-associated nucleotidyl-transferase (NiRAN)-like domain (Figure 1) [11].

Upon successful invasion of a host cell, the host’s protein synthesis machinery is hijacked to facilitate the translation of RdRp, utilizing the viral genomic RNA as a template [12,13]. RdRp assumes a critical role in orchestrating multiple vital processes, including the completion of negative-strand viral genome RNA transcription, the synthesis of various mRNA molecules encoding structural proteins, and the replication of viral genomic RNA [14,15,16]. Therefore, RdRp becomes a potential clinical target in therapeutic research and development efforts. Moreover, mammalian cells lack a counterpart to RdRp, and its inhibition does not result in side effects related to the target [17,18,19,20].

**Figure 2:**
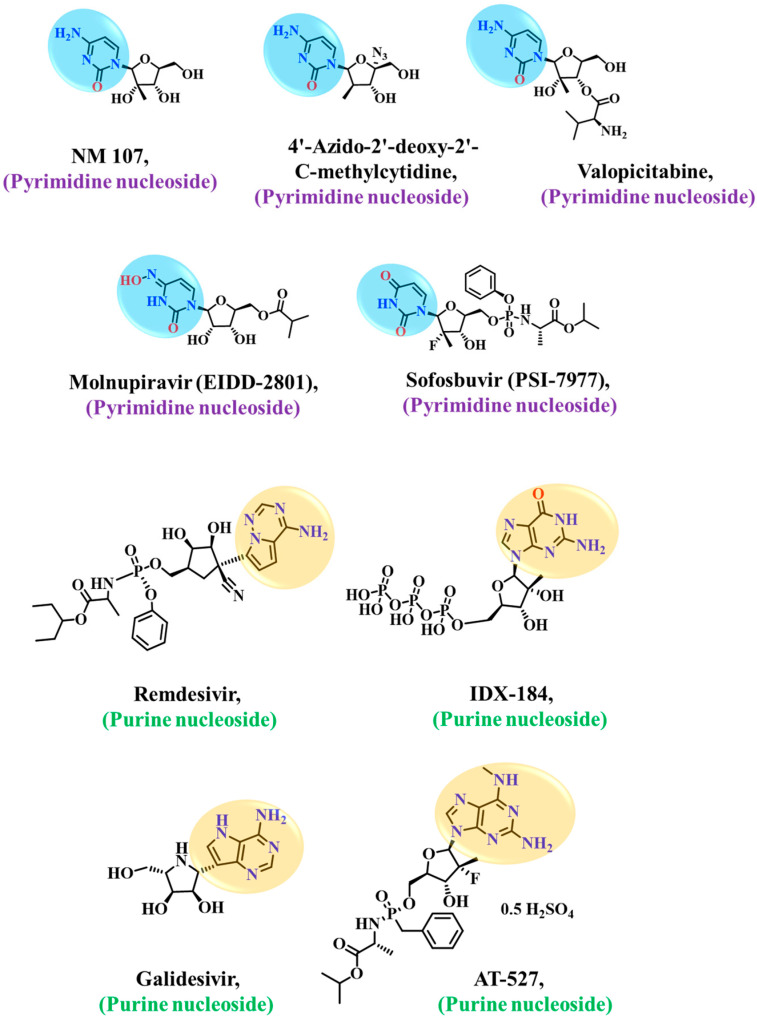
Chemical structures of reported nucleoside inhibitors of SARS-CoV-2 RdRp. The nucleobases are highlighted in blue (pyrimidine), and light orange (purine).

Consequently, targeting RdRp represents an indispensable strategy in the fight against SARS-CoV-2, aiming to impede virus replication. RdRp inhibitors can be divided into nucleoside inhibitors (NIs) and non-nucleoside inhibitors (NNIs) based on their structural characteristics. Nucleoside inhibitors can be incorporated into an elongating RNA chain and bind to the RdRp active center, leading to chain termination and inhibition of RdRp activity [21]. Remdesivir, Favipiravir, Molnupiravir, AT-527, etc., were repurposed as SARS-CoV-2 RdRp nucleoside inhibitors and tested for their anti-COVID-19 efficacy (Figure 2). Remdesivir (EC_50_ = 0.77 M) was the first drug approved by the FDA in 2020 to treat COVID-19. The reported analysis showed that Remdesivir is a prodrug transformed inside cells into triphosphate (RTP, active form). Gilead Sciences created monophosphorylated Remdesivir to have more excellent cellular absorption in vivo. Exoribonuclease (ExoN) proofreading efficiency is interfered with, and RdRp polymerization activity is inhibited by the GS-441524 1′-CN group, which is crucial in the termination of RNA replication [22]. Nevertheless, despite several clinical trials, Remdesivir has not shown a significantly earlier efficacy compared to the placebo group [23]. Furthermore, it has proven unsuccessful in significantly improving mortality, cure rate, recovery time, and other relevant indicators [24,25]. The main drawback of Remdesivir is that it can only be administered intravenously and is therefore logistically hard to use for therapeutic purposes at home, especially on a big scale [26]. In order to overcome the limitations of the reported nucleoside analog for the inhibition of RdRp, our current approach is based on the computational design of the nucleoside analogs to inhibit the function of SARS-CoV-2 RdRp.

**Figure 3:**
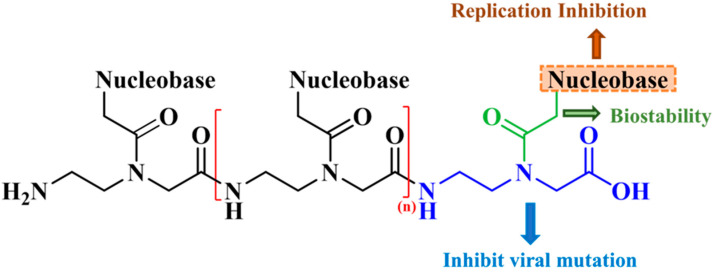
General structure of peptide nucleic acid (PNA) oligomer. PNA backbone (N-(2-aminoethyl) glycine) is shown in blue, and methylene carbonyl linkage attached to the nucleotide bases is shown in green. Closed brackets represent repeating PNA monomer unit in designed oligomer (n = 3 PNA monomers).

Peptide nucleic acids (PNAs) represent a class of single-stranded synthetic RNA/DNA analogs that feature a unique chemical composition. These molecules incorporate an uncharged and flexible N-(2-aminoethyl) glycine unit in place of the traditional phosphodiester backbone, while a methylene carbonyl linkage connects the nucleotide bases (Figure 3). This substitution imparts PNAs with distinct physicochemical properties, including low toxicity and remarkable biostability against peptidases and nucleases found in biological fluids [27,28]. The robust stability of PNAs enables their utilization as reliable tools in various research fields, facilitating the development of innovative diagnostic methods and targeted therapeutic interventions [29,30].

Our investigation revealed that there is no extensive research on the utilization of PNA oligomers as a crucial component in SARS-CoV-2 medication therapy. In recent research, Bichismita Sahu and coworkers designed scaffolds formed from PNA monomer and systematically performed in silico analysis. The designed compounds have good RdRp-binding affinity [31,32]. In this study, we employed computational simulation techniques to develop a novel leading compound by exploiting the mode of action on SARS-CoV-2 RdRp and modifying a newly designed peptide. Our primary focus lies on the nucleoside reverse transcriptase inhibitor PNA, with both RdRp and RdRp-RNA of SARS-CoV-2 serving as the targets. However, PNA analogs face challenges regarding their cellular uptake capability, necessitating an appropriate delivery method to enhance their therapeutic effects. Cell-penetrating peptides are frequently employed to transport therapeutic compounds directly to their target cells. Therefore, we modified the PNA backbone covalently coupled with cell-penetrating peptides to facilitate the efficient delivery of charge-neutral PNAs into cells. 

Moreover, the susceptibility of peptides to degradation by peptidases and proteases poses a significant concern. To enhance peptide stability, we incorporated D-amino acids into the designed PNA-peptide conjugates [33]. Via virtual screening, we identified that PNA analogs conjugated with cell-penetrating peptides (CPPs) might exhibit inhibitory activity against viral RdRp and the RdRp-RNA complex. This approach serves as a valuable model for the development of anti-SARS-CoV-2 candidate drugs. The newly designed PNA molecules can be paired with RNA bases that regulate the function of RdRp, inhibiting their integration into nucleic acids. Meanwhile, once inside the cell, these PNA molecules might target the RdRp enzyme and end the replication of RNA viruses by effectively inhibiting its activity and thereby achieving a dual-target impact.

## 2. Results 

### 2.1. Design Strategy of PNA Analogs

**Figure 4:**
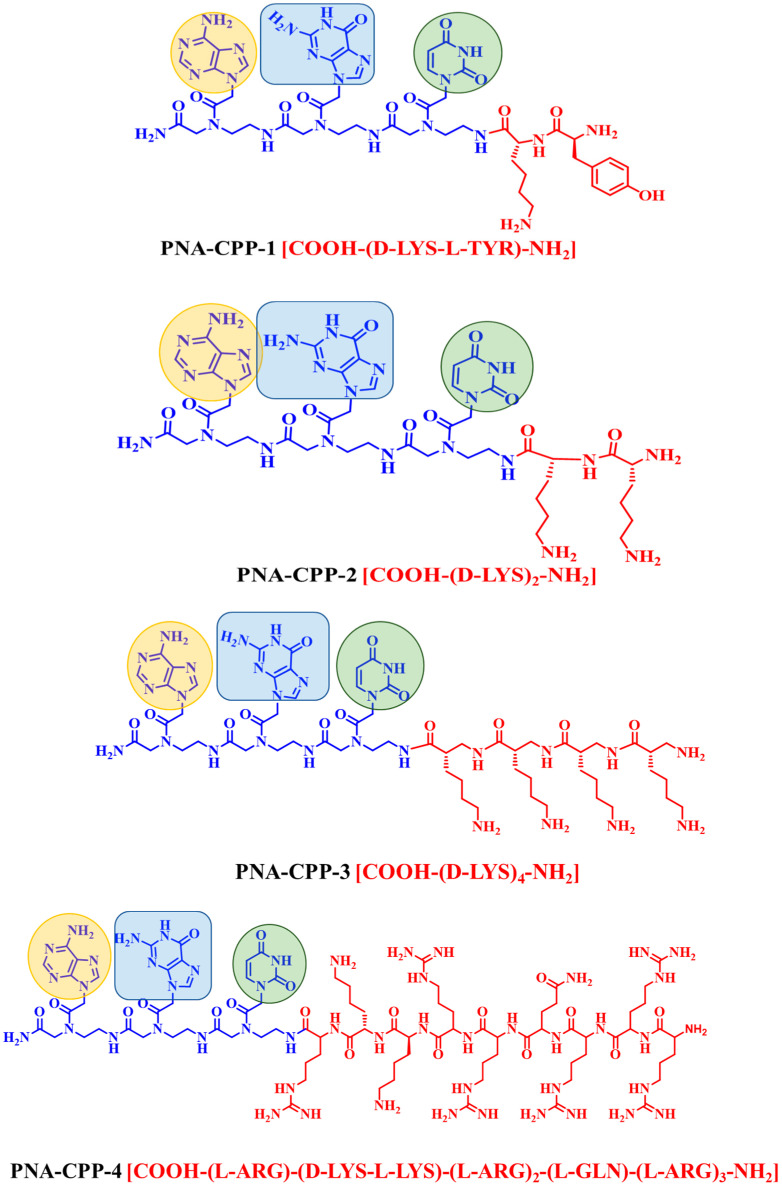

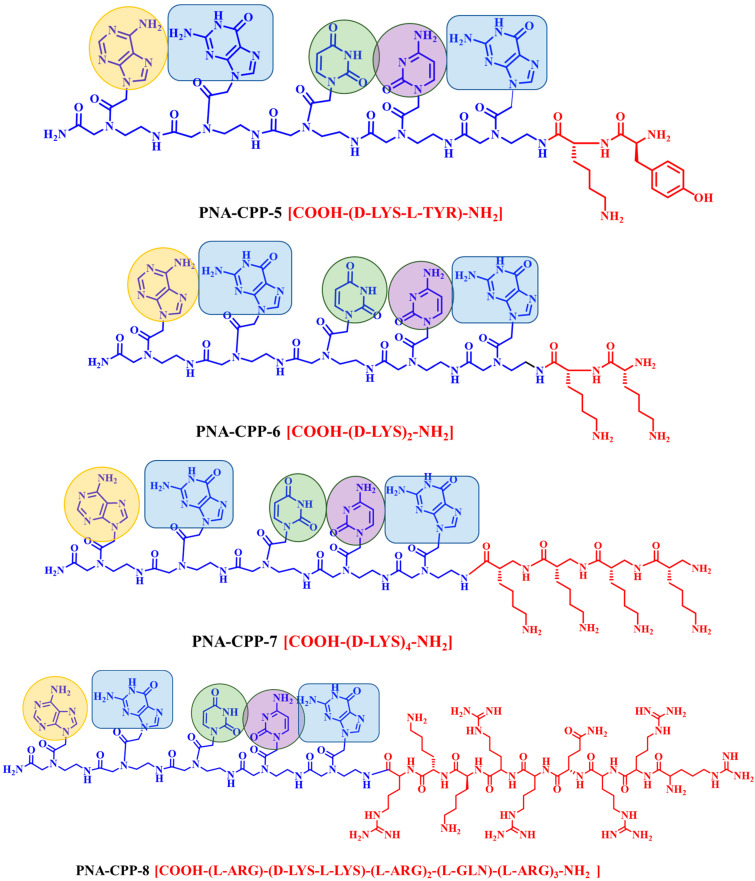
Chemical structures of designed PNA analogs. PNA oligomers (PNA-CPP-1 to 8) (colored in blue) covalently attached with CPP (colored in red). The nucleobases are highlighted in light orange (adenine), blue (guanine), light green (uracil), and purple (cytosine).

The chemical structures of PNAs were designed complementary to a targeted gene of SARS-CoV-2 RdRp. The target gene must first be determined to construct the initial structure of the PNA analog. The target RdRp regions correspond to the published SARS-CoV-2 genome (NCBI Reference Sequence: NC_045512.2 from 13,442 to 16,236 bp) (Figure S2). After obtaining the gene encoding the RdRp enzyme, we selected the first five bases of the gene to design the base position on the PNA monomer. We primarily use complementary base pairing, nucleic acid hybridization concepts, and artificial synthesis. The complementary fragments of the appropriate PNA are designed to interact with the target gene’s particular sequence, preventing the target gene’s replication and transcription. The electrostatically neutral backbone of PNA contributes to its exceptionally strong binding without compromising specificity. For efficient cellular uptake, PNAs were covalently linked to the small cell-penetrating peptide (CPP) (Figure 4). We initially designed a PNA oligomer comprising five complimentary nucleobases attached to two positively charged hydrophilic basic amino acids (D-lysine).

Further, these initially designed PNA oligomers were also attached with commercially available CPP (DK4 and HIV-1 TAT). Unfortunately, the docking scores were not so good. To improve the binding interaction, we shortened the length of the designed PNA and created a more stable PNA analog attached to DK4 and HIV-1 TAT CPP. The binding affinity of short-length PNA oligomer increases comparatively. However, the PNA oligomer with three nucleobases (AGU) linked with one positively charged hydrophilic basic amino acid (D-lysine) and one hydrophobic aromatic amino acid (L-Tyrosine) (PNA-CPP-1, Figure 4) exhibits strong binding interaction among all newly designed PNA analogs.

### 2.2. Analysis of Molecular Interactions by Molecular Docking

The designed PNA-CPP analogs were docked against all four predicted potentially druggable cavities of RdRp. They were ranked based on their binding affinities with the target residues. Remdesivir was analyzed as the reference ligand in comparison with other ligands by molecular docking. The optimal lead molecule (PNA-CPP-1) was selected based on its binding affinity and intermolecular interactions with key residues of RdRp compared to Remdesivir. Interestingly, we found that our best-docked compound PNA-CPP-1 shows strong binding interactions in all the predicted druggable cavities of RdRp protein compared to other designed compounds, and the amino acids residues involved in all predicted cavities are given in Table S2. The results indicate that PNA-CPP-1 binds more tightly in the potentially druggable cavity 4, thus impairing the polymerase function of RdRp of SARS-CoV-2 with a binding energy of −9.19 Kcal/mol. PNA-CPP-1 shows efficient binding interactions with the receptor protein via conventional hydrogen bonds, hydrophobic (π-π, alkyl-pi), and ionic interaction compared to others in all druggable cavities of the receptor protein. The binding energy and inhibition potential (Ki) values of the best lead are shown in Table 1. The binding energy suggests that PNA-CPP-1 would be selected as a leading candidate for RdRp synthetic inhibitor.

**Figure 5:**
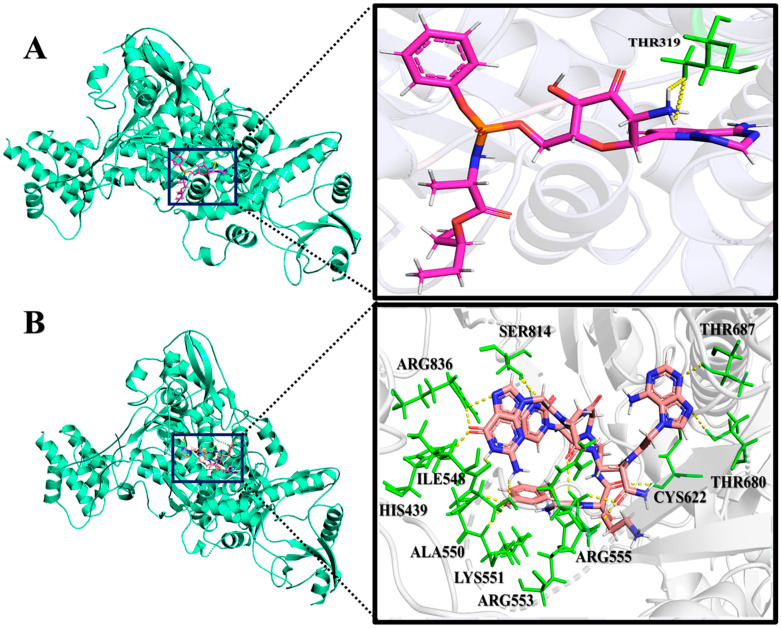
The interaction analysis of PNA-CPP-1 with SARS-CoV-2 RdRp. (**A**) Close-up view of 3D model of the interaction between Remdesivir and RdRp (PDB ID: 7BTF). (**B**) Close-up view of 3D interaction diagram of PNA-CPP-1 with RdRp (PDB ID: 7BTF). The cartoon model represents the catalytic part of the RdRp protein. Close-up PNA-CPP-1 (salmon color) and Remdesivir (purple color) bound at the same active sites of RdRp protein. Amino acids forming hydrogen bonds with ligands are colored green.

PNA-CPP-1 shows promising results of binding energy among the selected ligands because of its strong binding interactions (Figure S4) within all druggable binding cavities of the RdRp (Tables S3–S6). Interestingly, PNA-CPP-1 exhibits ten conventional hydrogen bonds within the druggable cavity 4 of RdRp. Furthermore, nine hydrophobic interactions are observed with amino acid residues of the RdRp (Table S6). The previously reported FDA-approved RdRp inhibitor Remdesivir was additionally docked into the predictive druggable cavity of RdRp protein to validate the predictive quality and accuracy of our de novo design approach. The docking analysis of the Remdesivir showed that it formed a hydrogen bond with THR319 a less binding affinity (−8.69 Kcal/mol) compared to our best-docked ligand PNA-CPP-1 (forming hydrogen bonds with HIS439, SER814, ALA550, THR680, THR687, ARG553, ARG555, CYS622, ILE548, and LYS551) (−9.19 Kcal/mol) (Figure 5A,B) in the same predicted druggable cavity.

**Figure 6:**
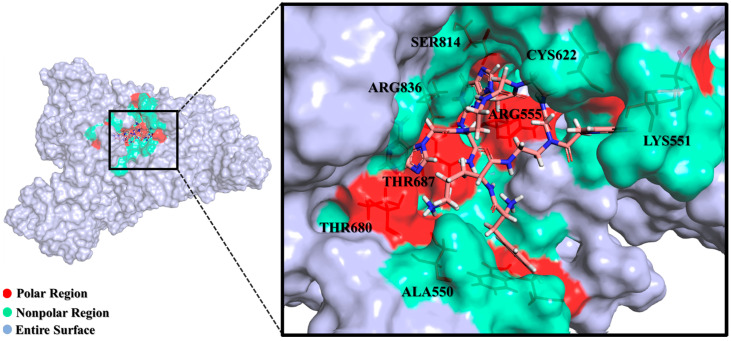
Surface model of the interaction between PNA-CPP-1 and RdRp (PDB ID: 7BTF). Closeup view of polar and non-polar interactions of best-docked ligand (PNA-CPP-1). Light blue area characterizes the entire surface of the protein, while red and cyan-colored regions illustrate polar and nonpolar areas, respectively. Closeup surface view indicates that PNA-CPP-1 (colored in salmon) forms conventional hydrogen bonds with key polar region residues of the binding pocket (colored in red stick).

It can be observed that hydrogen bonds and hydrophobic intermolecular interactions participate in major types of interactions in designed complexes. Moreover, it has been shown that PNA-CPP-1 has interacted with more polar residues than non-polar residues of the target protein (Figure 6), which indicates the importance of polar functional groups at this position, leading to a strong interaction between PNA-CPP-1 and RdRp. The computational analysis of the reported nucleotide-based RdRp inhibitor (Remdesivir, Favipiravir, etc.) showed that the free amine group of the triazine–pyrrole complex, amide functionality, and the hydroxyl group of pyrimidine scaffold interact with charged and polar amino acids residues of RdRp catalytic pocket, which indicated the importance of polar functional groups for the inhibitory activity of nucleotide-based ligands [34,35,36].

Furthermore, we also performed blind docking of PNA-CPP-1 With RdRp (PDB ID: 7BTF), which confirms that PNA-CPP-1 can also effectively bind at the experimentally determined binding site of RdRp (PDB ID: 7BTF) of SARS-CoV-2. The interaction analysis showed that PNA-CPP-1 forms a conventional hydrogen bond with key residues involved in the formation of the catalytic center and NTP entry channel, including ASP760, ASP761, LYS545, and ARG555 (See Figure S3).

In addition, the docking study with the RdRp-RNA complex predicts that PNA-CPP-1 can be effectively integrated into the growing RNA strand. The in silico analysis predicts that the PNA-CPP-1 molecule, which might act as a nucleotide analog by incorporating into the primer strand forms the interaction with the uridine base (U20) from the primer strand via two hydrogen bonds. Moreover, we hypothesized that the selected leading compound, PNA-CPP-1, might potentially inhibit the transcription of SARS-CoV-2 by intervening with RNA’s function on its growth site of RdRp. PNA-CPP-1 also interacts with the crucial amino acid residues LYS545 and ARG555, which help to maintain the incoming nucleotide in the proper place for catalysis (Figure S5). According to the literature, the RdRp’s catalytic active site interacts with the primer strand of RNA by contacting the +1 base with side chains of LYS545 and ARG555, stabilizing the incoming nucleotide in the proper position for catalysis. The computational analysis predicts that, like Remdesivir, leading PNA-CPP-1 blocks SARS-CoV-2 replication by forming hydrogen bonds with the side chain of LYS545 and ARG555. PNA-CPP-1 also interacts with SER682 and LYS545. These two residues are essential during the fidelity check before incorporation [37].

Furthermore, to support our hypothesis that PNA-CPP-1 can potentially inhibit the transcription of SARS-CoV-2 by intervening with RNA’s function on its growth site of RdRp, we first dock the natural nucleotide ATP with RdRp-RNA complex (PDB ID: 7BV2). The interaction analysis (Figure S6A,B) exhibits that ATP is incorporated into the primer strand binding with U20 (Uracil) and, according to the complementary base pairing rule, showed interaction with U10 (uracil) base of template strand with binding energy −6.86 Kcal/mol which is less than PNA-CPP-1 (−9.94 Kcal/mol) at the same binding site. The interaction analysis of ATP with the binding site of RdRp-RNA reveals that it showed a strong interaction with LYS545 and ARG555, which contributes to the formation of the NTP entry channel. Interestingly, when we dock the ATP with the RdRp-RNA-PNA complex, the binding affinity of ATP is further reduced to −5.89 Kcal/mol, and it is displaced from its position and also changes its confirmation. From the interaction analysis, we conclude that ATP cannot be stably incorporated into the primer strand due to the presence of PNA, which is attached to U20 (uracil) of the primer strand. Based on interaction analysis and binding affinity of ATP with RdRp-RNA with or without PNA (Figure S7A,B), we hypothesized that PNA could potentially mimic the purine (Adenine) base natural ATP counterpart of viral RdRp to form non-canonical base pairs and can cause delayed chain termination or it can potentially slow down RNA synthesis by viral RdRp.

**Figure 7:**
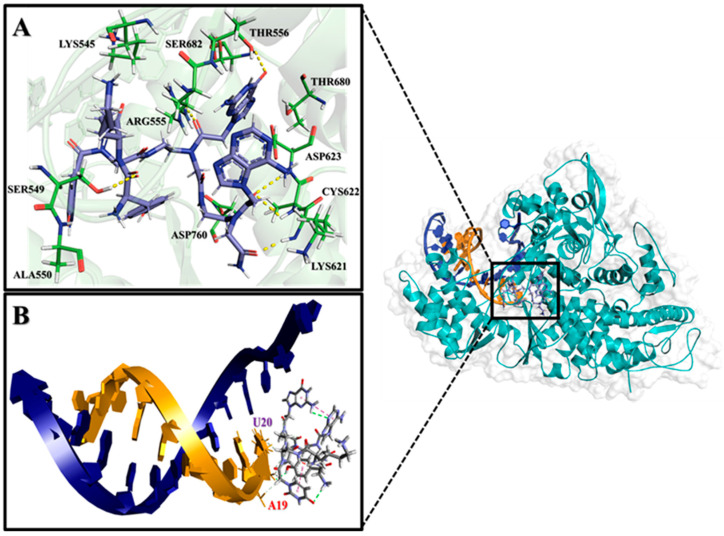
The interaction analysis of PNA-CPP-1 with SARS-CoV-2 RdRp-RNA (PDB ID: 7BV2). (**A**) Close-up view of the RdRp complex with RNA active site shows the interaction of PNA-CPP-1 with key amino acid residues. (**B**) PNA-CPP-1 is incorporated into the primer strand and might terminate chain elongation, showing key nucleotides (U20, A19) interacting with PNA-CPP-1.

Moreover, our docking analysis is validated by the reported computational analysis of Remdesivir with the RdRp-RNA complex (PDB ID: 7BV2) [38]. PNA-CPP-1 selected leading compound interact with catalytic key residues and nucleotide bases including, SER682, SER759, ASP760, ARG553, ASP623, THR687, ASN691, A19 (Adenine), and U20 (Uracil) (Figure 7). The binding affinity of the lead compound target RdRp-RNA complex (PDB ID: 7BV2) is presented in Table 2. Detailed non-bond information, interacting amino acids, and dihedral angle are provided in Table S7.

Commercial drugs might form H-bonds with essential residues of SARS-CoV-2 RdRp. The binding interaction analysis of Remdesivir exhibited strong hydrogen bonds with the key residues LYS545, ARG553, and ARG555 of the target receptor. Similarly, PNA-CPP-1 showed the highest binding affinity among the other designed PNA compounds by forming hydrogen bonds with LYS545, ARG553, and ARG555, along with other key amino acid residues, to inhibit the function of RdRp of SARS-CoV-2 [39]. Hydrogen bonds are crucial in determining ligand binding specificity to receptors, biological activity, and molecular recognition in chemical and biological drug design processes. The designed compounds tend to be surrounded by the previously mentioned residues, similar to the arrangement in standard drugs, according to the docking investigation of all the PNA analogs with the SARS-CoV-2 RdRp.

Moreover, we performed validation analysis via the superimposition of Remdesivir and PNA-CPP-1 complexes with both targets, including RdRp-RNA (PDB ID: 7BV2) and RdRp (PDB ID: 7BTF). The superimposition of both target complexes shows that PNA-CPP-1 has the same binding site as the experimentally determined binding site for Remdesivir (reference drug) to impair the polymerase activity of RdRp of SARS-CoV-2 (See Figures S8 and S9). This discovery revealed that these newly designed PNA analogs might prevent SARS-CoV-2 from replication by inhibiting the activity of RdRp.

### 2.3. Frontier Molecular Orbitals and Molecular Electrostatic Potential Analysis

The chemical quantum study investigated the chemical stability of the best-docked PNA-CPP-1 ligands, including the analysis of frontier molecular orbitals (FMO) and molecular electrostatic potential (MESP). The highest occupied molecular orbital (HOMO) and lowest unoccupied molecular orbital (LUMO) are commonly referred to as FMOs. The structural characteristics of the ligands employed for creating docking interactions with target protein receptors are essential in describing the density distribution pattern on frontier molecular orbitals [40]. The energetics of FMOs significantly affect the reactivities and chemical stability of ligands. We apply density functional theory at the B3LYP-D3/6-311+G (d,p) level to optimize and calculate the electronic and structural properties of the designed ligands. It has been shown that the higher HOMO energy and lower LUMO energy in the drug molecule might lead to larger stabilizing interactions of the ligand molecule with the target receptor [41].

**Figure 8:**
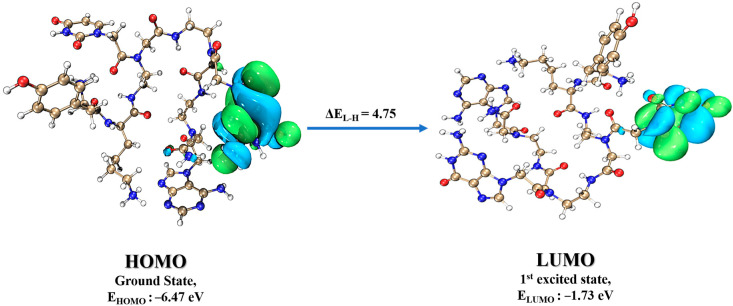
The FMOs, including HOMO and LUMO for PNA-CPP-1, calculated at the B3LYP-D3/6-311+G (d,p) level of DFT.

Moreover, the HOMO-LUMO energy difference is essential in determining the reactivity and stability of the molecule. A lower band gap suggests the molecule is less stable and more susceptible to electrophilic and nucleophilic attack. The FMOs analysis shows that the ΔE_L-H_ (eV) value of the HOMO-LUMO gap of PNA-CPP-1 is 4.75 eV, which offers the stability of PNA-CPP-1 (Figure 8).

The MESP map shows the complete charge of the nuclei and electrons. It also provides insight into the molecule’s dipole moment, electronegativity, chemical reactivity, and partial charge. Atomic charges are used to examine the correlation between the biological activity and chemical structure of drugs in computer-aided drug design. MESP is frequently employed as a reactivity map showing the most optimal places for charged-point-like substances to attack organic compounds in nucleophilic and electrophilic ways. MESP aids in the interpretation of hydrogen bond interactions and biological recognition mechanisms. It is an easy approach for predicting potential interactions between various geometries provided by the MESP counter map. Both the ligand and protein molecules have partial charges, which are essential when a ligand attaches to a protein in the docking process. The structural features of ligands are depicted in three dimensions by their molecular electrostatic potential. The total density surface’s point where the influence of nuclei or electrons is most pronounced can be found using MESP [42]. The 3D structural aspects are evaluated using MESP [43].

**Figure 9:**
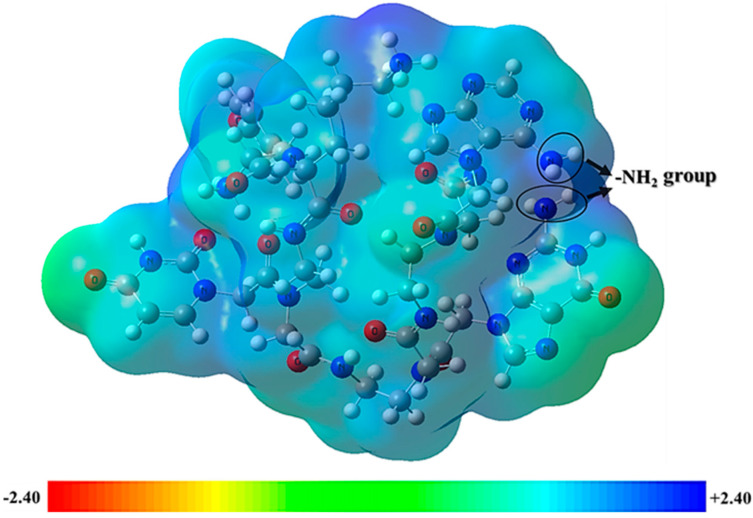
MESP plots for PNA-CPP-1 at B3LYP-D3/6-311+G (d,p) level of DFT with the iso-values of ±0.240. The blue and red regions display the positive and negative potentials of the ligands, respectively, while green highlights zero potential.

The MESP of PNA-CPP-1 was obtained at the B3LYP-D3/6-311+G (d,p) level of DFT with iso-values of ±0.240. The hydrogen atom has the most positive potentiality, while the oxygen atom has the highest negative potentiality, according to the MESP map. The optimized structure of the best docked PNA analog’s reactive sites for electrophilic and nucleophilic assaults was determined using MESP. The positive electrostatic potential is shown on the MESP diagrams in the blue portion, the green part shows the zero electrostatic potential, and the red portion shows the negative electrostatic potential (Figure 9). In our selected lead compound, more positive regions (blue) are mainly toward the protonic H-atom of amine groups (-NH_2_ and -NH of purine and pyrimidine ring), which will potentially act as H-bond donors in protein–ligand intermolecular interactions. The hydrogen bonds between acidic amine groups of nucleobases attached to PNA analogs and crucial amino acid residues (TRP268, SER397, ASN628, ARG349, ASP623, THR680, SER682, ARG553, ARG555, and CYS622) of RdRp and RdRp-RNA complex (see Figure 5B and Figure 7) are shown here in the blue area, which is the maximum positive area and preferable for nucleophilic attack. The previously published nucleotide-based RdRp research showed that the -NH_2_ of the nucleobase is needed for nucleophilic attack and is an indispensable functionality of the binding interactions with the essential amino acid of binding sites to inhibit RdRp function. Conversely, most negative regions (red) are around carbonyl groups owing to their electron abundance environment, which is also an attractive site for electrophilic attack. The carbonyl group of lead ligands involved in non-bond interaction with SER549, ASP623, LYS621, ALA550, SER397, and ASN628 residues of target receptor by electrophilic attack.

### 2.4. Global Chemical Descriptors (GCDs)

The energies of FMOs are correlated to the electron affinity and ionization potentials of molecular systems based on the band gap. The other significant properties, like ionization energy (I), chemical hardness, chemical softness, electronegativity, electrophilicity index, and electronic chemical potential, are estimated based on the HOMO and LUMO band gap [44]. Most of these aforementioned electronic structure properties are used in quantitative structure-activity relationship (QSAR) studies. We consider them worth calculating and present these factors as valuable information for our best-designed ligand (PNA-CPP-1).

The ionization potential value of the PNA-CPP-1 was 6.47 eV, which shows its thermal stability. The PNA analog’s chemical hardness (η) and softness (S) were 2.37 and 0.42, respectively, correlating with the energy band gap. A small energy band gap (ΔE_L-H_) exhibits softness, while the larger band gap (ΔE_L-H_) shows the hardness of the docked complex. As a result, the hard compound is stable, but the soft compound is more reactive and polarizable [45]. The electron affinity of PNA-CPP-1 was 1.73 eV, which shows that it can more readily accept electrons due to its higher LUMO value. The global chemical descriptors suggest the selected ligand possesses good stability and chemical strengths (Table 3).

### 2.5. Analysis of Molecular Dynamics Simulation Trajectory

The apo and holo state of RdRp (PDB ID: 7BTF) and RdRp-RNA (PDB ID: 7BV2) were simulated using YASARA software (YASARA Biosciences GmbH, Vienna, VIE, Austria, (accessed on 20 June 2023)). for 800 ns. To determine the stability and dynamics of each system, the RMSD, radius of gyration, and SASA were analyzed from the resultant molecular dynamic trajectories using YASARA built-in MD analysis macro “md_analysis.mcr”. The results of these simulations are compared using a wide range of parameters and observations after reaching a plausible equilibrium and are further described as follows:

### 2.6. Stability Analysis by RMSD

**Figure 10:**
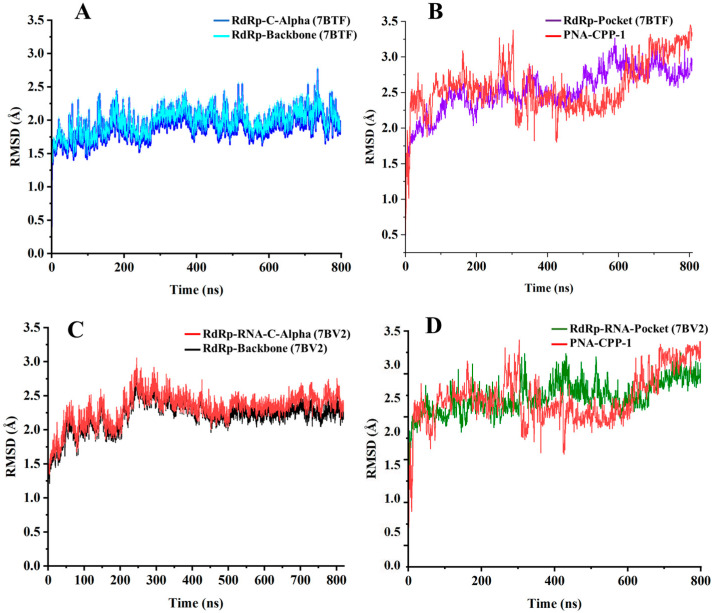
The RMSD profiles of 800 ns MD simulations of RdRp and RdRp-RNA with PNA-CPP-1. The structures included in simulations were RdRp protein (PDB ID:7BTF), RdRp-RNA (PDB ID: 7BV2), and PNA-CPP-1. (**A**) RMSD of the C-alpha of RdRp (7BTF) is in blue, and all backbone atoms are shown in cyan. (**B**) The RMSD of the pocket residues of RdRp (7BTF) is colored purple, and the RMSD of the unbound ligand is colored red. (**C**) RMSD of the C-alpha of RdRp-RNA (7BV2) is colored red, and all backbone atoms are colored black. (**D**) The RMSD of the pocket residues of RdRp-RNA (7BV2) is colored in green, and the RMSD of the ligand is shown in red.

The time evolution of the RMSD was calculated to verify the dynamic stability of the protein and ligand complex. The RMSD is an essential parameter to predict the dynamic stability of antiviral compounds in the receptor’s binding pocket. To analyze the conformational changes in protein backbone atom from its initial confirmation to its final position in free and complex states using 800 ns molecular dynamics simulations. The structural stability is significantly higher when the root mean square deviation is lowest. The catalytic pocket domain of protein exhibits a constant stable graph up to 500 ns. After 500 ns, it showed minor structural RMSD deviation from 500 to 600 ns and then became stable after 600 ns (Figure 10A). The RMSD of the pocket residues in the complex was reasonably stable according to interaction analysis within the active pockets of the protein molecule, compared with the RMSD of the unbound ligand and receptor (Figure 10B and Figure S14). The best-retrieved molecule (PNA-CPP-1) retained interactions with the catalytic amino acid’s residues, including LYS551, ARG553, TRP268, ARG555, THR556, LYS621, and ASN628 of RdRp, exhibited by pocket residues dynamic stability, indicating the inhibitory potential of the selected hit.

The critical amino acid residues of the catalytic pocket in RdRp-RNA (PDB ID: 7BV2) retained stability up to 800 ns (Figure 10D). However, the unbound form of PNA-CPP-1 showed minor fluctuations throughout the 800 ns molecular dynamic simulation. For PNA-CPP-1-RdRp, the protein backbone and C-alpha atoms have not deviated throughout 800 ns. Conversely, the PNA-CPP-1-RdRp-RNA complex showed minor fluctuation from 200 to 300 ns. After that, the simulation converged, and the system was equilibrated (Figure 10C). Furthermore, after a slight deviation, the system remained consistent throughout the 800 ns MD simulation and found no significant variation compared to the native structure. We can conclude that the interaction with PNA-CPP-1 can stabilize the structure of the SARS-CoV-2 RdRp protein with or without RNA. The RMSD graph of the protein and ligand reveals that it remained stable for the simulation period with only minor fluctuations.

### 2.7. Steered Molecular Dynamics Simulations

In our investigation of the stabilization of pocket residues upon ligand (PNA-CPP-1) binding to RdRp and RdRp-RNA, we conducted SMD simulations spanning 3 ns, applying vector forces of 6 pN, 8 pN, and 10 pN, respectively. After applying three different forces, the fluctuation in the distance of pocket residues in PNA-CPP-1 bound RdRp-RNA did not show much pronunciation. Under an applied force of 6 pN, 8 pN, and 10 pN, the distance between LYS545 and ARG555 remained confined within 12.59 Å. In contrast, the distances between SER682 and ASP761 exhibited variations of up to 2 Å via the application of three different vector forces. Concurrently, the PNA-CPP-1-bound complex displayed a decrease in the distance between ASN691 and ASP760 by approximately 4 Å to reach approximately 22 Å by applying 8 pN force, a change not observed in 10 pN force, which maintained a consistent distance of around 30 Å as in case of 6 pN force (See Figures S10 and S11). However, after applying three different forces, the distance between pocket residues of RdRp, including ALA550, LYS551, CYS622, THR680, ARG555, and ARG553, showed minor fluctuation. These simulations provide evidence suggesting that the PNA-CPP-1 confers not much more fluctuation to the binding pocket residues of RdRp and RdRp-RNA of SARS-CoV-2.

### 2.8. Radius of Gyration Analysis

The radius of gyration (Rg) values, i.e., the mass weight root means square distance for assembling atoms from their common centers of mass of the two systems, were calculated to examine the overall compactness of protein. A protein remains stable during the simulation when Rg exhibits a reasonably constant value throughout time. Graphs of the RdRp protein (PDB ID: 7BTF) and RdRp-RNA (PDB ID: 7BV2) complex’s radius of gyration vs. time were drawn to verify this. The Rg graph for the RdRp-RNA complex shows the overall compactness despite some initial fluctuations. The Rg maintains an average value of 30.74 Å throughout 800 ns until the end of the simulation timeframe. The plot of fluctuations in Rg (Å) against the time (ns) is presented in (Figure S12A,B). Herein, the Rg of protein (total and around the axis) was found in the range of 38.5 to 35.4 Å. The maximum Rg value for the receptor protein (RdRp) was around ~35.8 at 450 ns. The average radius of gyration for the RdRp-RNA protein complex was computed to be around ~31.2 Å during the whole molecular dynamic simulations. The maximum Rg value was recorded at approximately 31.51 Å. These findings revealed that the Rg values for both complexes remain stable compared to unbound receptor (Figure S15), depicting the highest structural compactness of both targets after binding with the ligand.

### 2.9. Solvent-Accessible Surface Area (SASA) Analysis

The solvent-accessible surface area of the ligand-binding site is essential for evaluating the changes in ligand–protein interaction. The surface area of a biomolecule next to a solvent is called SASA [46]. This study helps to clarify whether the protein structure and the ligand–protein complex behave hydrophobic or hydrophilic in solvents [47,48,49]. The surface area (nm^2^) accessed by solvents or water molecules inside the active site was further studied using the plots of SASA versus run time (ns) for the apo–protein (RdRp) and protein complex (RdRp-RNA) as well (Figure S13A,B).

We observed that the surface area for the RdRp-RNA (PDB ID: 7BV2) complex is 370.25–385.62 nm^2^ with an average of 377.93 nm^2^, which showed stability throughout the 800 ns simulation. In contrast, the solvent-accessible surface area for the RdRp protein complex (PDB ID: 7BTF) is 420.25–438.62 nm^2^, with an average of 429.43 nm^2^. The analysis of SASA revealed that the PNACPP-1-RdRp and PNA-CPP-1-RdRp-RNA complex showed greater stability when binding with PNA-CPP-1.

### 2.10. Comparison of PNA-CPP-1 Complex before and after MD Simulation

The MD simulation analysis (in comparison to the docking) uncovered numerous new aspects of various specific target–ligand contacts and their cooperation in light of the time-accumulated statistical data, confirming the general conclusions of docking. The molecular docking analysis of the selected lead compound (PNA-CPP-1) showed that it has a strong binding affinity with the catalytic site of targeted RdRp protein compared with the reference drug (Remdesivir) against RdRp of SARS-CoV-2.MD simulations verified the reliability of the models constructed from protein–ligand docking. Both methods gave similar complex structures. Quantitatively, the RMSD values of the structures from the MD and docking are small (Figure 10), indicating that the structural variations are minor, and that the ligand remains inside the binding pocket.

**Figure 11:**
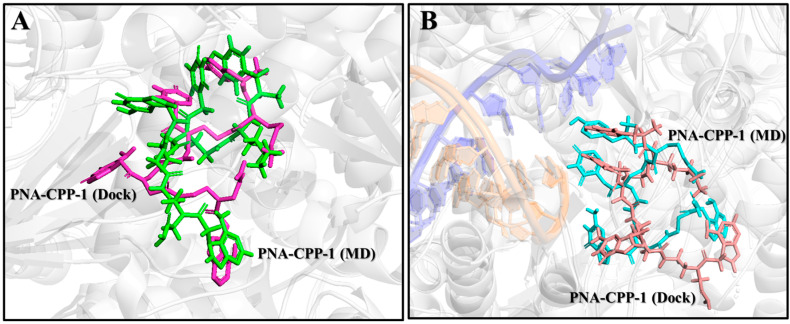
Superposition of two complexes, including the last frame obtained from the MD and the complex obtained from docking. (**A**) Superposition of RdRp (PDB ID:7BTF) and PNA-CPP-1 complex after docking (colored in purple) and after MD (colored in cyan). The RMSD value of systems aligned by PyMOL is 1.27. (**B**) Superposition of RdRp-RNA (PDB ID: 7BV2) and PNA-CPP-1 complex after docking (colored in cyan) and after MD (colored in blue). The RMSD value of the system aligned by PyMOL is 1.32.

Additionally, MD simulations of the PNA-CPP-1 complex with both targets, namely the RdRp and RdRp-RNA complex, show that it binds tightly to the SARS-CoV-2 polymerase protein via strong non-bonded interactions, which may inhibit the process of viral replication. Moreover, the structural superimposition of both PNA-CPP-1 complexes before and after MD simulation revealed no changes in overall conformation (Figure 11A,B). Also, after the simulation, the occupancy of PNA at the binding cavity of RdRp showed a slight shift in orientation, which appeared to be caused by a modification in the visible secondary structure at the PNA binding site. Furthermore, there was no significant fluctuation in the RMSD value for RdRp and RdRp-RNA, and it remained stable during the 800 ns MD simulation, proving that each ligand atom does not deviate so much from its original position. The docking analysis revealed that nucleoside analogs block the viral RdRp activity via polar interactions with receptor residues CYS619, LYS618, CYS622, ARG621, ARG550, and ARG553 [50].

**Figure 12:**
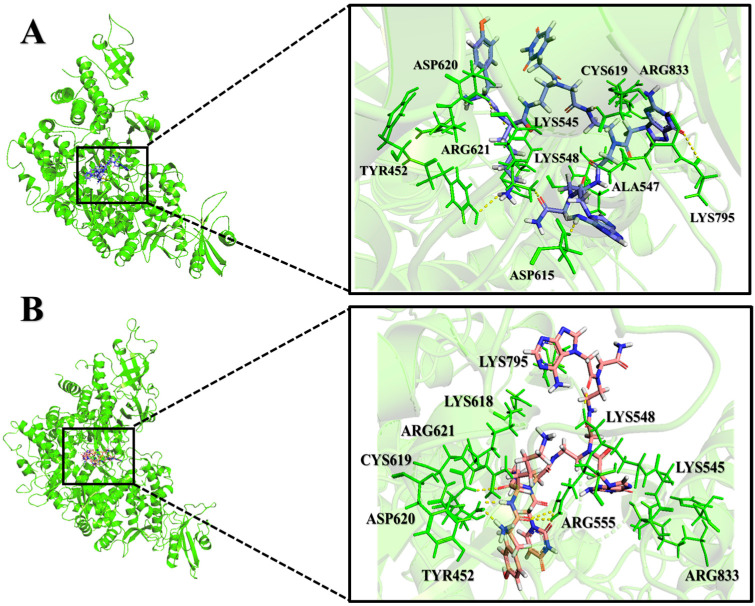
Interaction analysis of two complexes of PNA-CPP-1 with RdRp (PDB ID: 7BTF), including the initial and final structures obtained from MD. (**A**) PNA-CPP-1 complex and binding site residues (ligand colored in light blue) at 0 ns. (**B**) PNA-CPP-1 complex and binding site residues (ligand colored in salmon) at 800 ns. catalytic residue and other binding site residues are represented in green sticks. The cartoon model at the initial (**A**) and final (**B**) state of MD simulations depicted that PNA-CPP-1 remains in the binding cavity with minor fluctuations.

Moreover, we analyzed the trajectories obtained from the simulation at the initial and final pose of the PNA-CPP-RdRp complex (Figure 12A,B). It was observed that the PNA-CPP-1 strongly binds with the predicted druggable cavity 4 of RdRp. It interacts via hydrogen bonds with LYS618, ARG621, ARG550, ASP620, and LYS795 at 0 ns and maintains these interactions with catalytic residues throughout the simulation period and remains inside cavity 4. Thus, 800 ns MD simulations ensure that cavity 4 is the standard and suitable binding site of PNA-CPP-1 compared to other predicted druggable cavities. We also analyzed the initial and final pose of the RdRp-RNA-PNA-CPP-1 complex (Figure S16A,B). PNA-CPP-1 showed strong hydrogen bond interaction with ARG553, THR556, SER549, ALA550, and U20 (Uracil) at an initial 0 ns of MD simulations and maintained these interactions during the complete simulation period. We observed that PNA-CPP-1 increases the stability of targeted protein and validates the molecular docking results with the RMSD analysis we obtained with MD simulation. Overall, in accordance with the evaluation of the hypothesis, designed PNA analogs showed an adequate binding affinity with both targets RdRp and RdRp-RNA compared to Remdesivir (Figures S17 and S18), validating the proposed hypothesis’ accuracy. Future research on this selected lead compound (PNA-CPP-1) can be explored to inhibit viral replication, which will help to combat SARS-CoV-2.

Additionally, the evolution of interatomic distances between functional groups of lead ligand PNA-CPP-1 and the key amino acid residues of the binding site of RdRp during MD was also performed to obtain more detailed information (Figure S19). During the molecular dynamics’ simulation at 800 ns, from the plot of residue distances over time, it can be seen that the LYS621 and THR556 represented the distance between the O atom of the PNA backbone and the N atom of the pyrimidine ring, respectively. SER682 and ASP760 represented the distance between the O atom of the carbonyl group attached to the pyrimidine ring of guanine nucleobase and the -NH group of the PNA backbone, respectively. ARG555, ASP623, and CYS622 represented the distance between the O atom of the carbonyl group of the PNA backbone and the guanine moiety. ASN691 and THR680 represented the distance between the N atom of the imidazole ring of nucleobases. However, the distances between PNA-CPP-1 and two amino acid residues, LYS621 and THR556, showed variation during 800 ns MD simulations. This might explain why LYS621 and THR556 played a minor role in the binding of PNA-CPP-1 with RdRp. However, distances between the O atom of the carbonyl group attached with the pyrimidine ring of guanine nucleobase and -NH group of PNA backbone and key amino acid residues (SER682, ASP760, ARG555, ASP623, and CYS622) basically maintained the same during the MD simulation. All these MD results provide detailed information on the interactions between the PNA-CPP-1 and RdRp of SARS-CoV-2. Based on our calculations above, we show that the structural changes in the binding pocket are less pronounced when PNA-CPP-1 binds to RdRp, suggesting that PNA-CPP-1 could be a good inhibitor candidate against RdRp and therefore needs further experimental investigations.

### 2.11. In-Silico Pharmacokinetic Analysis

We observed the high binding affinity of PNA-CPP-1 with RdRp and RdRp-RNA; it should be interesting to explore the feasibility of developing PNA-CPP-1 into a therapeutic drug to prevent SARS-CoV-2 replication. We were interested in whether the selected lead PNA-CPP-1 is a potential druglike compound after observing its highly specific interaction and strong binding affinity with the targeted receptor compared to the reference drug (Remdesivir). The ADMET study revealed PNA physicochemical characteristics (Table 4). PNA-CPP-1 possesses a high TPSA (519.07 [Å]^2^) due to the more polar groups of hydroxyl and carbonyl oxygens as well as the amino and amine nitrogen compared to Remdesivir (213.36 [Å]^2^). Furthermore, the PNA-CPP-1 has an appropriate plasma protein binding (PPB = 79.26%) according to the estimated value <90%, which may lead to a relatively high therapeutic index like Remdesivir (PPB = 65.83%). Furthermore, the water solubility of PNA-CPP-1 (logS = −3.87) is greater than −5 like Remdesivir (logS = −3.05), reflecting its water solubility at 25 °C. The volume distribution value describes the drug-administered dose with the actual initial concentration in the circulation. It is a crucial parameter to describe the in vivo drug distribution. In computer-guided drug design, we can speculate the distribution characters for the designed compound according to its VD value, such as its condition binding to plasma protein, its distribution amount in body fluid, and its uptake amount in tissues. ADMETlab2.0 predicted that the PNA-CPP-1 has a higher volume distribution (VD = 0.76 L/kg) like Remdesivir (VD = 1.86 L/kg), presenting its more excellent distribution in blood plasma [51]. Additionally, the pharmacokinetic analysis indicated that PNA-CPP-1 would not penetrate the blood–brain barrier (BBB) like the reference drug (Remdesivir), suggesting it could be utilized safely without concern about potential neurotoxicity. However, the in silico analysis predicts that PNA-CPP-1 will not impact the five main cytochromes P450 (CYP) isoforms: CYP1A2, CYP2C19, CYP2C9, CYP2D6, and CYP3A4. The alteration in activity of these enzymes can disturb the pharmacokinetics of drugs [52]. The two most significant cytochrome P450 isoforms are CYPP2D6 and CYPP3A4. PNA-CPP-1 does not inhibit these enzymes, whereas CYP3A4 is inhibited by Remdesivir. Therefore, it can be inferred that PNA-CPP-1 could be safe when used in combination with other medications compared to Remdesivir. The results of the toxicity prediction showed that PNA-CPP-1 exhibits negative AMES toxicity. In contrast, Remdesivir is AMES-positive and can also induce significant cytotoxic effects [53], whereas in silico toxicity prediction showed negative mutagenicity and carcinogenicity responses of PNA-CPP-1 compared to Remdesivir. However, ADMETlab2.0 predicted that PNA-CPP-1 shows moderate toxicity like Remdesivir in the rat oral acute toxicity result, which is connected to acute mammalian toxicity.

Furthermore, via the Pfizer rule, compounds with logP (≤3) and high TPSA (≥75) are likely to be non-toxic [54]. The synthetic accessibility score (SA score) is predicted to estimate the ease of synthesis of the druglike compound based on a combination of fragment contributions and a complexity penalty, and compounds with SA score ≤ 6 might be easy to synthesize [55]. ADMETlab2.0 predicted that PNA-CPP-1 has no inhibitory impact on any hepatic cytochrome enzyme like Remdesivir; hence, it neither causes hepatotoxicity nor induces liver injury [56]. Therefore, it can be a potential drug for COVID-19 treatment. Another key benefit is that PNA-CPP-1 would primarily not cause teratogenic effects, which is supported by the fact that chemical compounds must have molecular weights below 500 Dalton and high lipophilicity to penetrate the placenta [57]. PNA-CPP-1 is a high molecular weight polar compound with logP equal to −1.81 compared to Remdesivir with logP 2.06 and has less molecular weight. Therefore, PNA-CPP-1 might be administered to pregnant women with SARS-CoV-2 infection without risk when compared to Remdesivir, which has no penetration and a substantial concentration across the placenta. Regarding medicinal chemistry friendliness, Remdesivir has one warning for the Brenk rule (see Table 4), whereas PNA-CPP-1 has no alertness for PAINS or the Brenk rule. The overall drug-likeness and physicochemical results of the lead compound PNA-CPP-1 indicate that it can be developed as an oral drug with further manipulation of the structure or the formulation. Thus, PNA-CPP-1 is predicated as a drug-likeness compound by ADMETlab2.0 via the Pfizer Rule and SA score.

### 2.12. Proposed Synthetic Route for PNA-CPP-1

**Figure 13:**
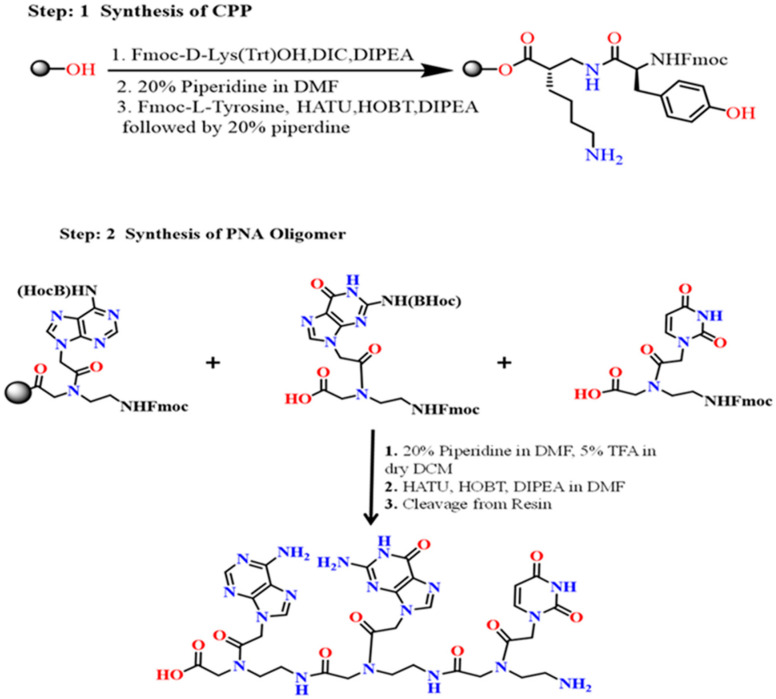

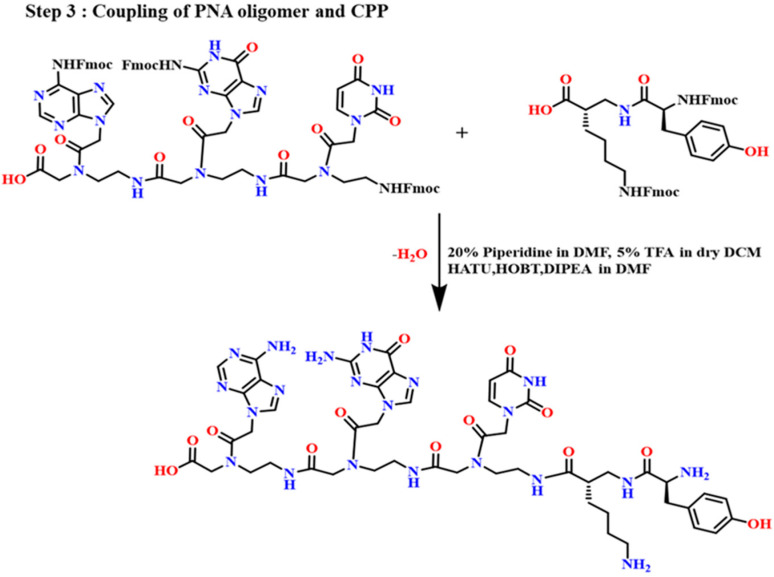
Proposed synthetic route for PNA-CPP-1.

We also designed the proposed synthetic route for the best-selected lead compound (PNA-CPP-1) in three steps. The PNA-CPP-1 consisted of D-Lysine and L-Tyrosine as the cell-penetrating peptide (CPP). PNAs with a small CPP peptide can be synthesized in the solid phase. NovaSynTGA resin (Merck, Darmstadt, Germany, 0.25 mmol/g) will be used as the solid support material. After adding the last amino acid, the protecting group will be removed by treating the resin-bound with 20% piperidine in DMF. The introduction of the PNA sequence to the peptide on the solid support, the first amino acid on the C-terminus (N-α-Fmoc-N-ε-trityl-D-lysine), was used as a handle. The trityl group was selectively deprotected using 5% (*v*/*v*) trifluoroacetic acid (TFA) in dry DCM, followed by the addition of Fmoc-PNA monomers via treatment with HATU (6 equiv.), HOBt (6 equiv.), and DIPEA (10 equiv.) in 0.15 M DMF for 3 h. In the last step, the cleavage of PNAs from resin will be performed. The peptide-PNA conjugates will be deprotected and released from the resin (as a free acid on the C-terminus) via treatment with 90:10 (*v*/*v*) TFA/m-cresol for the CPP-PNAs for 3 h (Figure 13). The PNA conjugates were triturated with cold ethyl ether, and the precipitate was collected. The rational development of the anti-coronavirus peptide nucleic acid conjugated with cell-penetrating peptide lead molecule and its proposed synthetic scheme will enable researchers in this field to develop a new generation of RdRp inhibitors with improved potency and specificity.

## 3. Discussion

The SARS-CoV-2 outbreak has devastated the naïve human population and is still causing havoc with newer waves of infections in various nations. In the current study, a computer-aided drug design strategy utilizing molecular simulation in conjunction with quantum chemicals assisted in identifying potential antagonists (PNA analogs) targeting RdRp and RdRp-RNA of SARS-CoV-2. Using the de novo drug design approach, eight nucleotide analogs covalently attached with CPP were designed, which have not been found for their inhibitory potential against RdRp of SARS-CoV-2 in any previous work. Each designed PNA-CPP was docked into the four predicted substantial druggable cavities of RdRp protein (PDB ID: 7BTF). Based on strong binding affinity in predicted druggable cavity four compared to other cavities, PNA-CPP-1 was selected as a top-hit ligand. To validate the docking results of PNA-CPP-1 in the standard binding cavity, molecular dynamics (MD) simulations were performed. This indicates that the druggable cavity 4 is a suitable binding site for the selected best-docked compound PNA-CPP-1. The PNA-CPP-1 was also docked with the RdRp-RNA complex (PDB ID: 7BV2). It was observed that PNA-CPP-1 is bound to U20 (Uracil) at the RNA-primer binding site with a strong affinity.

Docking analysis reveals that the previously reported nucleoside analogs might block the RdRp activity in viruses by working with the chain termination mechanism to form strong hydrogen bonds with crucial receptor residues, including LYS545, ARG550, ARG553, and ARG555 [58,59]. Interestingly, PNA-CPP-1 might also impede the entrance of NTP to the active site like Remdesivir by interacting with the LYS545, ARG553, and ARG555 residues in the NTP entry site via conventional hydrogen bonds.

Analysis of ADMET parameters of PNA-CPP-1 has shown favorable pharmacokinetic as well as pharmacodynamic characteristics exhibiting a high safety margin compared to Remdesivir against SARS-CoV-2. Overall, the ADMET analysis revealed that the PNA-CPP-1 has good absorption, volume distribution, and low toxicity, which might show PNA-CPP-1 as a potential candidate inhibitor of RdRp against SARS-CoV-2. Furthermore, both servers (ADMETlab2.0 and SWISS ADME) predicted that the SA score of PNA-CPP-1 is 5.41 (SA score ≤ 6), which makes it a fact that these compounds might be easy to synthesize [60]. Therefore, we also designed the proposed synthetic route for best hit compound (PNA-CPP-1) to pave the way for other researchers in this field to synthesize it with improved potency and efficacy. The process of discovering new drugs is a challenging, expensive, and complex process with an extremely low success rate. Recent developments in the field of computer-aided drug design have fueled the development of novel strategies to speed up the drug discovery process. We hope that our work will serve as some supporting evidence for other research groups working on the synthesis of nucleotide-based drugs to achieve PNA analogs, with the parts of our designed PNA allowing working in the active binding pocket of the targeting proteins to favor intracellular conformations, as guided by this computationally determined mechanism and proposed synthetic route.

Despite the rational design of a potential nucleotide-based inhibitor for SARS-CoV-2 RdRp, further optimization for the proposed hit compound should be performed before reaching the clinic investigation. However, the current study revealed that the PNA-CPP-1 position is at the center of the catalytic active site like other nucleoside analogs (Remdesivir, Molnupiravir, etc.). As a nucleobase analog, it can form base-stacking interactions with an upstream base from the primer strand and also showed interaction with the uridine base from the template strand. In addition, it showed strong interactions with RNA recognition residues (ASN691, SER682, and ASP623). Residues involved in the formation of catalytic center and NTP entry channel (ASP760, ASP761, LYS545, and ARG555) would ensure the destabilization of the incoming RNA in the active site and could potentially halt with the process of viral replication by inhibiting the RdRp of SARS-CoV-2. MESP analysis predicts that the protonic hydrogen atom of amine groups (-NH_2_ and -NH of purine and pyrimidine ring) will potentially act as H-bond donors in protein–ligand intermolecular interactions. These results will indicate that the structures of PNA-based drugs might be an indispensable functionality of the binding interactions with the essential amino acid of binding sites to inhibit RdRp function.

## 4. Materials and Methods

### 4.1. Preparation of Receptors and Ligands

The 3D crystal structure of RdRp in apo form (PDB ID: 7BTF) and in complex with template-primer RNA (PDB ID: 7BV2) was obtained from the protein data bank (https://www.rcsb.org/ (accessed on 20 May 2023)). The 7BTF is a complex with full-length nsp12 (RdRp), nsp7, and nsp8 of the SARS-CoV-2, while 7BV2 is a full-length nsp12-nsp7-nsp8 complex bound to the fifty-base template-primer RNA and the triphosphate form of Remdesivir (RTP) [61]. We used two structures of SARS-CoV-2 RdRp (PDB IDs: 7BTF and 7BV2) in our study. 7BTF is in reduced form and 7BV2 is co-crystallized with the inhibitor Remdesivir, allowing the precise identification of the nucleoside binding sites (orthostatic). We superimposed two structures to check their similarity (Figure S1). The overall structural architecture of the apo-RdRp (without Remdesivir, PDB ID: 7BTF) and complex-RdRp (with Remdesivir, PDB ID: 7BV2) is similar, except that nsp12 is in the closed conformation in apo-RdRp. The binding of nsp7 and nsp8 stabilizes the closed conformation. Furthermore, the target protein was optimized using Discovery Studio v4.5 (BIOVIA Corp, San Diego, CA, USA, (accessed on 20 May 2023)). During the preparation and optimization process, all the water molecules and associated cofactors (nsp7–nsp8) were removed from the protein molecule. Chain A (nsp12) was selected for further analysis; the missing atoms of the incomplete amino acid sidechain or backbone and the hydrogen atoms were added to the 3D structures of protein using Discovery Studio v4.5. Furthermore, the structure of two targeting proteins, RdRp (PDB ID: 7BTF) and RdRp-RNA (PDB ID: 7BV2) files, was prepared using the structure clean module of YASARA (accessed on 22 May 2023), such as removing crystallographic waters, adding polar hydrogens, and assigning charges to titratable amino acids [62] using default docking simulation macro “dock_run.mcr” using an AMBER03 force field, and geometry optimization using the steepest gradient approach (100 iterations). Protonation states of amino acids were generated at pH 7.4 by using the default pH option in YASARA to simulate physiological conditions. To remove bumps and ascertain the covalent geometry of the PNA-CPPs ligands, the RdRp structures were all energy-minimized via the NOVA force field [63].

The structures of the designed ligands were built by ChemBio3D Ultra (accessed on 20 May 2023) [64]. The generated SDF file format of all compounds was converted into a 3D PDB file format using Discovery Studio v4.5. The optimized forms of designed PNA-CPP analogs were obtained after energy minimization by Discovery Studio v4.5. The energy minimization was performed using CHARMm force fields until the RMS (Root Mean Square) gradient descent of 0.1 Kcal/(mol × Å) was reached. Moreover, ligands were energy-minimized before docking using the NOVA force field (YASARA software) [63] to eliminate bumps and obtain molecules with consistent energy values by removing any potential steric conflicts of the ligands. Simulated annealing was used to continue the process once conformational stress was removed using a brief steepest descent minimization until convergence. The ligands with the lowest energy were stored in the PDB format for further molecular docking analysis.

### 4.2. Prediction of the Binding Site of the Receptor

**Figure 14:**
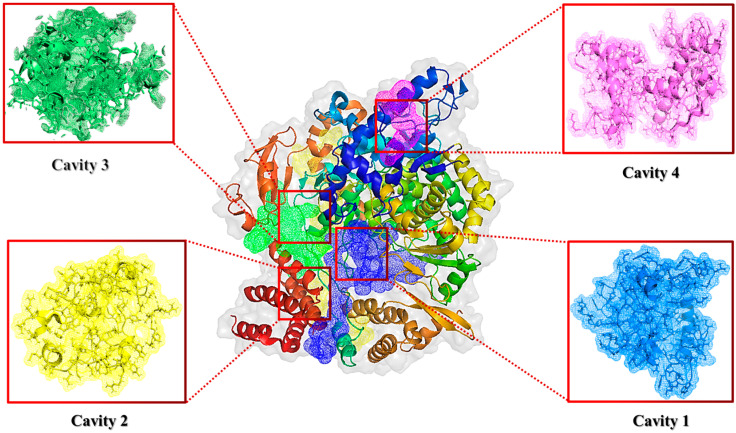
Potential drug-binding cavities of RdRp detected by Cavity Plus. The druggable cavities are represented as cavities 1, 2, 3, and 4.

The strong druggable binding pockets and catalytic sites of RdRp (PDB ID: 7BTF) were predicted using the CASTp server (Computed Atlas of Surface Topography of Proteins; http://cast.engr.uic.edu (accessed on 21 May 2023) [65], which provides the surface area and volume of binding pockets. The server identifies a putative binding pocket with a solvent-accessible surface area of 2943 Å^2^ and volume of 5705 Å^3^. Additionally, the online web server for protein cavity detector CavityPlus (www.pkumdl.cn:8000/cavityplus/computation.php (accessed on 21 May 2023) was also used to predict the potentially druggable cavities and performed several functional evaluations [66]. These two online web servers predict similar residues in the potential druggable binding pocket, with minor differences. The CavityPlus web server revealed that RdRp (nsp12) of SARS-CoV-2 contains four substantial druggable cavities, as shown in Figure 14. By using the CavityMatch module of the CavityPlus web server, we confirm the predicted binding site with the described binding sites in the literature. As per the knowledge of the binding site of the RdRp, it is prudent to examine the interaction among these predicted druggable target residues with the designed ligands.

### 4.3. Molecular Docking

The molecular docking studies of the designed PNA-CPP analogs against target proteins (RdRp and RdRp-RNA of SARS-CoV-2) were performed utilizing Autodock VINA in YASARA (YASARA Biosciences GmbH, Vienna, Austria) [67]. The ligand structures were all energy-minimized with the NOVA force field to remove the bumps and determine the covalent geometry of the ligands. After that, the receptor grid box in the size of 62.07 Å × 62.07 Å × 62.07 Å was generated around predicted druggable cavity 1 of RdRp (PDB ID: 7BTF); 55.58 Å × 55.58 Å × 55.58 Å around the conserved residues of predicted Cavity 2; 57.55 Å × 57.55 Å × 57.55 Å for druggable cavity 3; and 47.29 Å × 47.29 Å × 47.29 Å around the druggable cavity 4. Moreover, to target the RdRp-RNA, Remdesivir was used as a referenced ligand to build the grid box before docking analysis. The grid box was set in 34.08 Å × 34.08 Å × 34.08 Å. Then, the docking studies on the designed PNAs were carried out on YASARA via the built-in docking simulation macro “dock_run.mcr” using an AMBER03 force field with 25 poses and 9 clusters for each situation.

### 4.4. Interaction Analysis and Visualization of the Receptor–Ligand Complex

After a molecular docking analysis, the interactions between the designed PNA and the target protein were investigated using the “Ligand Interactions” feature in the “Receptor-Ligand Interactions” module of the Discovery Studio v4.5. The bond distances, dihedral angles, the types of non-bond interactions, and amino acid residues were labeled to characterize and visualize ligand binding regions inside the protein. Furthermore, the structural results were examined and shown using several software tools, including PyMoL [68] and Discovery Studio v4.5.

### 4.5. Quantum Chemical Analysis

The chemical quantum study investigated the chemical stability of the best-docked PNA-CPP-1 ligands, including the analysis of frontier molecular orbitals (FMO) and molecular electrostatic potential (MESP). The pharmacological impact of the drug molecule was found to be connected to its electronic properties [69]. We performed additional computational analyses using density functional theory (DFT) and molecular electrostatic potential (MESP) iso-surface analysis to acquire physical insights into the binding affinity pattern of the ligand molecules with RdRp. The DFT analysis was performed to examine the electronic structure properties of the top-hit ligand molecule. The theoretical chemical quantum calculations for the molecular structural analysis were performed using the GAUSSIAN 09 suit (https://gaussian.com, accessed on 10 June 2023) [70,71]. In contrast, orbitals and molecular electrostatic potential MESP frontier molecular orbitals were visualized using Multiwfn and VMD software (accessed on 11 June 2023, sobereva.com/multiwfn/download.html, https://softradar.com/vmd/) [72,73]. For quantum chemical calculations of ligand structures, their chemical structures were optimized (the lowest energy conformation) using gradient-corrected (density functional theory) DFT with the three-parameter hybrid functional (B3) [74] for the exchange part. The Lee–Yang–Parr (LYP) correlation function [75] was utilized to compute the molecular structure, vibrational frequencies, and energies of the optimized structures [76]. Moreover, to further explain the dispersion interactions that the B3LYP function is unable to describe, B3LYP-D3 was employed [77]. Meanwhile, the basis set 6-311+G (d,p) was augmented by polarization functions on heavy atoms, polarization functions on hydrogen atoms, and diffuse functions for both hydrogen and heavy atoms [78]. Optimized geometries were used to calculate the HOMO and LUMO energy parameters in this study.

### 4.6. Molecular Dynamics Simulations

To examine whether the fluctuation and conformational changes in the model constructed from protein–ligand docking are reliable, a molecular dynamics (MD) simulation of the binding between the SARS-CoV-2 RdRp, RdRp-RNA, and the selected PNA-CPP-1 was performed using YASARA software [67]. The YASARA dynamic software package (www.yasara.org/downloads.htm) [79] conducted MD simulation using the AMBER14 force field [80]. Each protein–ligand complex was subject to hydrogen bond optimization prior to dynamic simulation. The electrostatic interactions, particularly the long range, were calculated using the particle mesh Ewald method (PME) [81]. The initial energy minimization process was conducted using the steepest gradient algorithms’ simulated annealing method. The periodic boundary conditions were incorporated to handle long-range Coulomb forces over an 8 Å cutoff distance to calculate the Coulomb and van der Waals interactions. The TIP3P solvation model was used in a cubic simulation cell with a periodic boundary condition [82]. NaCl was employed at a concentration of 0.9%, the pH was 7.4, and the MD cell’s water density was 0.997 g/mL. No restraints were applied during the MD simulation using the settings employed in the second equilibration dynamics. In the NVT ensemble, the pressure was left uncontrolled. At the same time, energies and coordinates were stored every 100 ps at a constant temperature of 298 K. Finally, the NPA ensemble was used to implement the production run for 800 ns at a temperature of 298 K and a pressure of 1 bar. This work examined the molecular dynamics of PNA, PNA-RdRp, and PNA-RdRp-RNA complex using the Nosé–Poincaré–Andersen (NPA)—the most accurate and sensitive algorithm—thermostat to maintain a constant temperature of 298 K. Finally, the entire production run for MD simulations was carried out for 800 ns, followed by root means square deviation (RMSD) of Cα atoms, all backbone atoms of protein, and pocket interaction analysis conducted on MD trajectory files.

Furthermore, the structural stability of the protein–ligand complex and compactness of the protein secondary structure upon binding with the ligand were examined by analyzing RMSD values, the radius of gyration (Rg), and solvent accessible surface area (SASA). The RMSD profiles of all MD structures (Figure 10) show that the variation in the RMSD values tends to be stable after the long-range dynamic simulation, which means that the equilibrium structures have been obtained. The lowest energy MD simulation frame was chosen for later analysis by using YASARA via the built-in macro “md_analysis.mcr”. All these analyses confirmed ligand retention inside the binding pocket, and no unfolding in protein structure was observed over the explored time scale.

### 4.7. Steered Molecular Dynamics Simulations

SMD simulation [83] is an enhanced sampling molecular dynamics simulation that investigates how a molecule or protein responds to an external force by applying a direction vector to it. The 3 ns of SMD production runs were performed after 720 ps unconstrained MD simulation. First, the cubic tank was resized to a structural size of 10 Å using VMD/NAMD2. Energy minimization and MD simulations were performed at 720 ps using NAMD2 (version 2.14, accessed on 25 November 2023) employing the CHARMM36 force field [84]. The minimization and equilibration of the system were performed in three steps: (1) energy minimization of water molecules, counterions, and amino acid side chains (25,000 steps); (2) energy minimization of the whole system at a temperature of 310 K (25,000 steps); and (3) MD simulations were run at constant pressure (720,000 steps). Finally, a 720 ps MD simulation was performed. The temperature was kept at 310 K, and the Langevin dynamics of all non-hydrogen atoms were used with a Langevin damping factor of 1 ps^−1^. The pressure was kept at 1 atmosphere using a Nosé–Hoover–Langevin piston [85]. The simulations were performed with periodic boundary conditions, and remote electrostatics were calculated using the Ewald particle mesh method. The SHAKE algorithm constrained hydrogen bonding. SMD simulations were subsequently performed with the Cα atoms of ARG555, SER682, and ASP760 held stationary while external forces were applied to the centers of mass of the Cα atoms of LYS545, ASP761, and ASN691 for RdRp-RNA-PNA-CPP-1 complex, whereas for RdRp-PNA-CPP-1 ALA550, CYS622 and ARG555 held stationary while external forces were applied to the centers of mass of the C α atoms of LYS551, THR680, and ARG553. The direction of the constant force (6 pN, 8 pN, and 10 pN) was defined by the vector connecting the centers of mass of the Cα atoms. The other settings were consistent with the previous MD, with the difference that the SMD was run for 3 ns after the end of the 720 ps MD, and the time step used in the SMD production simulations was 2 fs. The VMD software was used to analyze the change in the distances of the binding pocket residues in the entire SMD trajectory.

### 4.8. Binding Free Energy Analysis

In this study, to validate our analysis, re-docking along with MMPBSA analysis of lead PNA-CPP-1 was performed by using Schrödinger’s GLIDE (accessed on 28 May 2023) [86] and YASARA to confirm further the binding free energy and affinity of the top-hit ligand. The results showed that the PNA-CPP-1 was found to have the highest binding energy of all in silico tools. PNA-CPP-1 was found to have a much higher binding affinity than the reference drug Remdesivir (Table S1). The molecular mechanics Poisson–Boltzmann surface area (MMPBSA) [87,88] method was used to calculate the binding free energies of both complexes, including RdRp-PNA-CPP-1 and RdRp-RNA-PNA-CPP-1. The calculations were conducted using YASARA software. The simulation trajectory was given to the default macro scripts of YASARA dynamics structure binding energy with an 800 ns time frame. The snapshots from the complete 800 ns MD simulation were used for both PNA-CPP-1 complexes. This analysis aims to estimate the final binding energy per 0.25 ns interval of RdRp-PNA-CPP-1 and RdRp-RNA-PNA-CPP-1 complexes in Kcal/mol per 0.5 ns interval at 800 ns simulation time. According to MMPBSA analysis, compound PNA-CPP-1 shows significant binding affinity and forms a stable complex with RdRp and RdRp-RNA, respectively. Protein–ligand binding free energy values were calculated using the following equation:

∆G_binding_ = ∆G_complex_ − [∆G_ligand_ + ∆G_protein_],

∆G_binding_ = ∆G_MM_ + ∆G_PB_ + ∆G_SA_ − T_∆S_ = (∆G_elec._ + ∆G_VdW_) + ∆G_PB_ + ∆G_SA_ − T_∆S_

where ∆G_complex_ = total free energy of the protein–ligand complex in the solvent.

∆G_ligand_ = total energy of the ligand in the solvent.
∆G_protein_ = total energy of the protein in solvent.
∆G_MM_ = molecular mechanics interaction energy, where the ∆G_elec._ and ∆G_VdW_ are the electrostatic and Van der Waals interactions, respectively.
∆G_PB_ and ∆G_SA_ represent polar solvation and non-polar solvation energy, respectively.
T_∆S_ (temperature = T and entropy = S) is the contribution of entropy to the free energy.

The average binding free energy of the PNA-CPP-1-RdRp complex throughout the simulation was −32.27 Kcal/mol. However, the average binding free energy of RdRp-RNA bound with PNA-CPP-1 during 800 ns MD simulation was observed at −92.06 Kcal/mol. This observation highlights that PNA-CPP-1 developed better intermolecular contacts (hydrogen bond and hydrophobic interactions) with the key residues of target RdRp and RdRp-RNA, securing the top-rank binding free energy (Figure S20A,B).

### 4.9. ADMET Study

Physicochemical and structural analysis is crucial for successful drug discovery in the pharmaceutical industry. ADMET (absorption, distribution, metabolism, excretion, and toxicity) analysis is essential for effective computational drugs and minimizing side effects. In the current investigation, the online database server ADMETlab2.0 (https://admetmesh.scbdd.com/service/evaluation/index, (accessed on 20 August 2023)) was used to evaluate the potential draggability of best docked PNA analog via ADMET analysis. ADMETlab2.0 uses several high-quality prediction models trained using a multi-task graph attention framework to conveniently and effectively employ the computation and prediction of the pharmacokinetics, physicochemical properties, drug chemical friendliness, and drug-likeness of the leading compounds.

The canonical SMILES of the designed PNA-CPP compounds were copied using ChemBioDraw Ultra software (version 15.0, accessed on 20 May 2023) for ADMET analysis. The structure of the PNA analogs is then used to represent the parameters, such as physicochemical qualities, medicinal chemistry measurements, and ADMET behaviors. We also used the two other web servers, including pkCSM [89] (http://structure.bioc.cam.ac.uk/pkcsm (accessed on 21 August 2023)) and SWISS ADME [90] (http://www.swissadme.ch (accessed on 21 August 2023)), to validate the prediction of the ADMET properties by ADMETlab2.0 [60]. Online servers provide verifiable information for determining physicochemical characteristics, including pharmacokinetics, water solubility, lipophilicity, toxicity, drug-likeness, etc. The “Lipinski’s Rule of Five”, which illustrates the oral bioavailability of chosen ligands, was used to assess the drug-likeness of various compounds [91].

## 5. Conclusions and Future Aspects

In this study, we rationally designed PNA analogs that might both exhibit the unique ability to effectively bind within the catalytic druggable binding cavity of RdRp and integrate into the RNA growth site. The chemical structures of PNAs were designed complementary to a targeted gene of SARS-CoV-2 RdRp. We selected the first five nucleobases of the gene to attach with the base position on the PNA, primarily based on the complementary base pairing concept. The challenge of poor cellular permeability inherent to PNAs was strategically tackled by incorporating a small penetrating peptide into the designed compound. Notably, the results of molecular docking and MD simulation studies reveal that PNA-CPP-1 stands out as a top-ranking ligand among these analogs, which forms strong interactions with the key amino acids and shows high binding affinity toward SARS-CoV-2 RdRp and RdRp-RNA.

Interestingly, the ADMET analysis of PNA-CPP-1 showed favorable pharmacokinetic and pharmacodynamic characteristics, exhibiting minimal toxicity and a high safety margin compared to Remdesivir against SARS-CoV-2. Therefore, PNAs might be considered a potential inhibitor of RdRp after clinical study. The integration of computer-aided research emerges as a cost-effective strategy for the logical development of novel drug candidates.

## Figures and Tables

**Table 1:** Molecular docking results of PNA-CPP in all four substantial druggable cavities of SARS-CoV-2 RdRp (PDB ID:7BTF).

Nucleobase Sequence	CPP	Compounds	Druggable Cavities	Binding Energy (Kcal/mol)	Ki Values (µmol)
AGU	COOH-KY-NH_2_	PNA-CPP-1	Cavity 1	−8.03	1.29
			Cavity 2	−7.96	1.45
			Cavity 3	−8.29	0.82
			Cavity 4	−9.19	0.18
AGU	COOH-KK-NH_2_	PNA-CPP-2	Cavity 1	−7.48	3.23
			Cavity 2	−7.59	2.69
			Cavity 3	−7.80	1.91
			Cavity 4	−7.22	5.06
AGU	COOH-dK4-NH_2_	PNA-CPP-3	Cavity 1	−6.37	21.19
			Cavity 2	−7.40	3.73
			Cavity 3	−7.33	4.22
			Cavity 4	−6.70	12.14
AGU	COOH-RRRQRRKKR-NH_2_	PNA-CPP-4	Cavity 1	−7.59	2.73
			Cavity 2	−6.58	14.92
			Cavity 3	−6.48	17.57
			Cavity 4	−6.91	8.60
AGUCG	COOH-KY-NH_2_	PNA-CPP-5	Cavity 1	−8.70	0.41
			Cavity 2	−8.13	1.29
			Cavity 3	−8.29	0.82
			Cavity 4	−8.21	0.95
AGUCG	COOH-KK-NH_2_	PNA-CPP-6	Cavity 1	−8.23	0.92
			Cavity 2	−7.65	2.46
			Cavity 3	−8.09	1.17
			Cavity 4	−7.79	1.94
AGUCG	COOH-RRRQRRKKR-NH_2_	PNA-CPP-7	Cavity 1	−7.09	6.33
			Cavity 2	−7.65	2.44
			Cavity 3	−8.00	1.36
			Cavity 4	−8.30	0.82
AGUCG	COOH-dK4-NH_2_	PNA-CPP-8	Cavity 1	−7.07	6.53
			Cavity 2	−7.14	5.83
			Cavity 3	−6.67	12.86
			Cavity 4	−6.39	20.63
		Remdesivir (Reference drug)	Cavity 1	−8.14	1.07
			Cavity 2	−7.65	2.45
			Cavity 3	−7.61	2.61
			Cavity 4	−8.69	0.42

A = Adenine; G = Guanine; C = Cytosine; U = Uracil.

**Table 2:** Molecular docking results of PNA-CPP-1 and Remdesivir with SARS-CoV-2 RdRp-RNA (PDB ID: 7BV2).

Compound	Binding Energy (Kcal/mol)	Inhibition Constant (µmol)	Residues Involved in H-Bond Interaction
PNA-CPP-1	−9.94	0.051	SER549, ALA550, ARG555, ARG553, LYS621, CYC622, ASP623, THR680, SER682, ASN691, ASP760, THR556, SER549, LYS545, SER681
Remdesivir	−7.92	1.54	LYS545, SER682, ASN691, ARG553, ARG555

**Table 3:** The calculated GCD results of PNA-CPP-1 at B3LYP-D3/6-311+G (d,p) level of DFT.

Parameters	PNA-CPP-1
E_HOMO_ (eV)	−6.47
E_LUMO_ (eV)	−1.73
ΔE_L-H_ (eV)	4.75
Ionization Energy (I) (eV)	6.47
Electron Affinity (A)	1.73
Chemical Hardness (η)	2.37
Chemical potential (μ)	−4.10
Electronegativity index (χ)	4.10
Electrophilicity index (ω)	6.68
Softness (S) (e^−1^V^−1^)	0.42

**Table 4:** Physicochemical and pharmacokinetic properties of PNA-CPP-1 and Remdesivir.

Properties	PNA-CPP-1	Remdesivir
Formula	C_63_H_74_N_22_O_14_	C_27_H_35_N_6_O_8_P
Fraction Csp3	0.35	0.48
Num. H-bond acceptors	20	12
Num. H-bond donors	12	4
Molar Refractivity	353.62	150.43
Topological Polar Surface area, TPSA ([Å]^2^)	519.07	213.36
LogP	−1.81	2.06
Water solubility (logS) (mol/L)	−3.87	−3.05
GI-absorption	moderate	moderate
P-gp substrate	Yes	Yes
P-glycoprotein I/II inhibitor	Yes	Yes
Log VD_ss_ (log L/Kg)	0.76	1.86
CYP2C19 inhibitor	No	No
CYP2C9 inhibitor	No	No
CYP2D6 substrate	No	No
CYP3A4 substrate	No	Yes
CYP2D6 Inhibitor	No	No
CYP3A4 Inhibitor	No	Yes
Rat Oral Acute Toxicity	moderate	moderate
Renal OCT2 substrate	No	No
Acute Toxicity Rule	0 alert	0 alert
Human hepatotoxicity (H-HT)	No	No
Skin sensitivity	No	No
Plasma protein binding (PPB)	79.26%	65.83%
Blood–brain barrier (BBB) permeability	No	No
SA score	5.418	4.815
AMES	No	Yes
PAINS	0 alert	0 alert
Brenk	0 alert	1 alert: Phosphorus
Bioavailability Score	0.17	0.17
Pfizer Rule	Accepted	Accepted

## Data Availability

The data supporting this work are available in this paper, and details are present in the supporting information.

## Supplementary Materials

The supporting information can be downloaded at https://www.mdpi.com/article/10.3390/ijms242417473/s1.

## Abbreviations

PNA	Peptide Nucleic Acid
CPP	Cell Penetrating Peptide
DIPEA	N, N-Diisopropylethylamine
RdRp	RNA-dependent RNA-Polymerase
HOBt	Hydroxybenzotriazole
HATU	Hexafluorophosphate Azabenzotriazole Tetramethyl Uronium
NTP	Nucleotide Triphosphate

## References

[1] Wu C., Liu Y., Yang Y., Zhang P., Zhong W., Wang Y., Wang Q., Xu Y., Li M., Li X. (2020). Analysis of therapeutic targets for SARS-CoV-2 and discovery of potential drugs by computational methods. Acta Pharm. Sin. B.

[2] Allan M., Lièvre M., Laurenson-Schafer H., de Barros S., Jinnai Y., Andrews S., Stricker T., Formigo J.P., Schultz C., Perrocheau A. (2022). The World Health Organization COVID-19 surveillance database. Int. J. Equit. Health.

[3] Hegelund M.H., Fjordside L., Faurholt-Jepsen D., Christensen D.L., Bygbjerg I.C. (2023). Opportunistic non-communicable diseases in times of COVID-19. APMIS.

[4] Shereen M.A., Khan S., Kazmi A., Bashir N., Siddique R. (2020). COVID-19 infection: Emergence, transmission, and characteristics of human coronaviruses. J. Adv. Res..

[5] Luttens A., Gullberg H., Abdurakhmanov E., Vo D.D., Akaberi D., Talibov V.O., Nekhotiaeva N., Vangeel L., De Jonghe S., Jochmans D. (2022). Ultralarge Virtual Screening Identifies SARS-CoV-2 Main Protease Inhibitors with Broad-Spectrum Activity against Coronaviruses. J. Am. Chem. Soc..

[6] Thakkar R., Agarwal D.K., Ranaweera C.B., Ishiguro S., Conda-Sheridan M., Gaudreault N.N., Comer J. (2023). De novo design of a stapled peptide targeting SARS-CoV-2 spike protein receptor-binding domain. RSC Med. Chem..

[7] Cao L., Goreshnik I., Coventry B., Case J.B., Miller L., Kozodoy L., Chen R.E., Carter L., Walls A.C., Park Y.-J. (2020). De novo design of picomolar SARS-CoV-2 miniprotein inhibitors. Science.

[8] Gao S., Huang T., Song L., Xu S., Cheng Y., Cherukupalli S., Kang D., Zhao T., Sun L., Zhang J. (2022). Medicinal chemistry strategies towards the development of effective SARS-CoV-2 inhibitors. Acta Pharm. Sin. B.

[9] Mousavi S., Zare S., Mirzaei M., Feizi A. (2022). Novel drug design for treatment of COVID-19: A systematic review of preclinical studies. Can. J. Infect. Dis. Med. Microbiol..

[10] Pundir H., Joshi T., Pant M., Bhat S., Pandey J., Chandra S., Tamta S. (2022). Identification of SARS-CoV-2 RNA dependent RNA polymerase inhibitors using pharmacophore modelling, molecular docking and molecular dynamics simulation approaches. J. Biomol. Struct. Dyn..

[11] Alanagreh L.A., Alzoughool F., Atoum M. (2020). The human coronavirus disease COVID-19: Its origin, characteristics, and insights into potential drugs and its mechanisms. Pathogens.

[12] de Farias S.T., Junior A.P.D.S., Rêgo T.G., José M.V. (2017). Origin and evolution of RNA-dependent RNA polymerase. Front. Genet..

[13] White K.A., Enjuanes L., Berkhout B. (2011). RNA Virus Replication, Transcription and Recombination.

[14] Venkataraman S., Prasad B.V., Selvarajan R. (2018). RNA dependent RNA polymerases: Insights from structure, function and evolution. Viruses.

[15] Ferrer-Orta C., Ferrero D., Verdaguer N. (2015). RNA-dependent RNA polymerases of picornaviruses: From the structure to regulatory mechanisms. Viruses.

[16] Bartas M., Volná A., Beaudoin C.A., Poulsen E.T., Červeň J., Brázda V., Pečinka P. (2022). Unheeded SARS-CoV-2 proteins? A deep look into negative-sense RNA. Brief. Bioinform..

[17] Shehzadi K., Saba A., Yu M., Liang J. (2023). Structure-Based Drug Design of RdRp Inhibitors against SARS-CoV-2. Top. Curr. Chem..

[18] Te Velthuis A.J. (2014). Common and unique features of viral RNA-dependent polymerases. Cell. Mol. Life Sci..

[19] Raj K., Kaur K., Gupta G., Singh S. (2021). Current understanding on molecular drug targets and emerging treatment strategy for novel coronavirus-19. Naunyn-Schmiedeberg’s Arch. Pharmacol..

[20] Jukič M., Janežič D., Bren U. (2021). Potential novel thioether-amide or guanidine-linker class of SARS-CoV-2 virus RNA-dependent RNA polymerase inhibitors identified by high-throughput virtual screening coupled to free-energy calculations. Int. J. Mol. Sci..

[21] Tian L., Qiang T., Liang C., Ren X., Jia M., Zhang J., Li J., Wan M., YuWen X., Li H. (2021). RNA-dependent RNA polymerase (RdRp) inhibitors: The current landscape and repurposing for the COVID-19 pandemic. Eur. J. Med. Chem..

[22] Li Y., Cao L., Li G., Cong F., Li Y., Sun J., Zhang X. (2021). Remdesivir metabolite GS-441524 effectively inhibits SARS-CoV-2 infection in mouse models. J. Med. Chem..

[23] Spinner C.D., Gottlieb R.L., Criner G.J., López J.R.A., Cattelan A.M., Viladomiu A.S., Ogbuagu O., Malhotra P., Mullane K.M., Castagna A. (2020). Effect of remdesivir vs standard care on clinical status at 11 days in patients with moderate COVID-19: A randomized clinical trial. JAMA.

[24] Wang Y., Zhang D., Du G., Du R., Zhao J., Jin Y., Fu S., Gao L., Cheng Z., Lu Q. (2020). Remdesivir in adults with severe COVID-19: A randomised, double-blind, placebo-controlled, multicentre trial. Lancet.

[25] Goldman J.D., Lye D.C., Hui D.S., Marks K.M., Bruno R., Montejano R., Spinner C.D., Galli M., Ahn M.-Y., Nahass R.G. (2020). Remdesivir for 5 or 10 days in patients with severe COVID-19. N. Engl. J. Med..

[26] Dallocchio R., Dessì A., De Vito A., Delogu G., Serra P., Madeddu G. (2021). Early combination treatment with existing HIV antivirals: An effective treatment for COVID-19?. Eur. Rev. Med. Pharmacol. Sci..

[27] Nielsen P.E. (2010). Peptide nucleic acids (PNA) in chemical biology and drug discovery. Chem. Biodivers..

[28] Nielsen P.E., Egholm M., Berg R.H., Buchardt O. (1991). Sequence-selective recognition of DNA by strand displacement with a thymine-substituted polyamide. Science.

[29] Gupta A., Mishra A., Puri N. (2017). Peptide nucleic acids: Advanced tools for biomedical applications. J. Biotechnol..

[30] Ahn D.-G., Lee W., Choi J.-K., Kim S.-J., Plant E.P., Almazán F., Taylor D.R., Enjuanes L., Oh J.-W. (2011). Interference of ribosomal frameshifting by antisense peptide nucleic acids suppresses SARS coronavirus replication. Antivir. Res..

[31] Sahu B., Behera S.K., Das R., Dalvi T., Chowdhury A., Dewangan B., Shard A. (2022). Design and in-silico screening of Peptide Nucleic Acid (PNA) inspired novel pronucleotide scaffolds targeting COVID-19. Curr. Comput.-Aided Drug Des..

[32] Park S., Kim S.H., Dezhbord M., Lee E.H., Jeon Y., Jung D., Kim K.H. (2023). Cell-permeable peptide nucleic acid antisense oligonucleotide platform targeting human betacoronaviruses. Front. Microbiol..

[33] Zorzi A., Deyle K., Heinis C. (2017). Cyclic peptide therapeutics: Past, present and future. Curr. Opin. Chem. Biol..

[34] Sada M., Saraya T., Ishii H., Okayama K., Hayashi Y., Tsugawa T., Nishina A., Murakami K., Kuroda M., Ryo A. (2020). Detailed molecular interactions of favipiravir with SARS-CoV-2, SARS-CoV, MERS-CoV, and influenza virus polymerases in silico. Microorganisms.

[35] Nguyen H.L., Thai N.Q., Truong D.T., Li M.S. (2020). Remdesivir strongly binds to both RNA-dependent RNA polymerase and main protease of SARS-CoV-2: Evidence from molecular simulations. J. Phys. Chem. B.

[36] Khan F.I., Kang T., Ali H., Lai D. (2021). Remdesivir strongly binds to RNA-dependent RNA polymerase, membrane protein, and main protease of SARS-CoV-2: Indication from molecular modeling and simulations. Front. Pharmacol..

[37] Wang Y., Li P., Solanki K., Li Y., Ma Z., Peppelenbosch M.P., Baig M.S., Pan Q. (2021). Viral polymerase binding and broad-spectrum antiviral activity of molnupiravir against human seasonal coronaviruses. Virology.

[38] Shannon A., Fattorini V., Sama B., Selisko B., Feracci M., Falcou C., Gauffre P., El Kazzi P., Delpal A., Decroly E. (2022). A dual mechanism of action of AT-527 against SARS-CoV-2 polymerase. Nat. Commun..

[39] Yin W., Mao C., Luan X., Shen D.-D., Shen Q., Su H., Wang X., Zhou F., Zhao W., Gao M. (2020). Structural basis for inhibition of the RNA-dependent RNA polymerase from SARS-CoV-2 by remdesivir. Science.

[40] Uengwetwanit T., Chutiwitoonchai N., Wichapong K., Karoonuthaisiri N. (2022). Identification of novel SARS-CoV-2 RNA dependent RNA polymerase (RdRp) inhibitors: From in silico screening to experimentally validated inhibitory activity. Comput. Struct. Biotechnol. J..

[41] Al Sheikh Ali A., Khan D., Naqvi A., Al-Blewi F.F., Rezki N., Aouad M.R., Hagar M. (2020). Design, synthesis, molecular modeling, anticancer studies, and density functional theory calculations of 4-(1, 2, 4-Triazol-3-ylsulfanylmethyl)-1, 2, 3-triazole derivatives. ACS Omega.

[42] Zheng Y., Zheng M., Ling X., Liu Y., Xue Y., An L., Gu N., Jin M. (2013). Design, synthesis, quantum chemical studies and biological activity evaluation of pyrazole–benzimidazole derivatives as potent Aurora A/B kinase inhibitors. Bioorganic Med. Chem. Lett..

[43] Kuruvilla T.K., Prasana J.C., Muthu S., George J., Mathew S.A. (2018). Quantum mechanical and spectroscopic (FT-IR, FT-Raman) study, NBO analysis, HOMO-LUMO, first order hyperpolarizability and molecular docking study of methyl [(3R)-3-(2-methylphenoxy)-3-phenylpropyl] amine by density functional method. Spectrochim. Acta Part A Mol. Biomol. Spectrosc..

[44] Shafieyoon P., Mehdipour E., Mary Y.S. (2019). Synthesis, characterization and biological investigation of glycine-based sulfonamide derivative and its complex: Vibration assignment, HOMO–LUMO analysis, MEP and molecular docking. J. Mol. Struct..

[45] Zia M., Muhammad S., Bibi S., Abbasi S.W., Al-Sehemi A.G., Chaudhary A.R., Bai F.Q. (2021). Exploring the potential of novel phenolic compounds as potential therapeutic candidates against SARS-CoV-2, using quantum chemistry, molecular docking and dynamic studies. Bioorganic Med. Chem. Lett..

[46] Adole V.A. (2021). Computational Chemistry Approach for the Investigation of Structural, Electronic, Chemical and Quantum Chemical Facets of Twelve Biginelli Adducts. Organomet. Chem..

[47] Mazola Y., Guirola O., Palomares S., Chinea G., Menéndez C., Hernández L., Musacchio A. (2015). A comparative molecular dynamics study of thermophilic and mesophilic β-fructosidase enzymes. J. Mol. Model..

[48] Khan R.J., Jha R.K., Amera G.M., Jain M., Singh E., Pathak A., Singh R.P., Muthukumaran J., Singh A.K. (2021). Targeting SARS-CoV-2: A systematic drug repurposing approach to identify promising inhibitors against 3C-like proteinase and 2′-O-ribose methyltransferase. J. Biomol. Struct. Dyn..

[49] Marsh J.A., Teichmann S.A. (2011). Relative solvent accessible surface area predicts protein conformational changes upon binding. Structure.

[50] Sahu S.N., Mishra B., Sahu R., Pattanayak S.K. (2022). Molecular dynamics simulation perception study of the binding affinity performance for main protease of SARS-CoV-2. J. Biomol. Struct. Dyn..

[51] Wakchaure P.D., Ghosh S., Ganguly B. (2020). Revealing the Inhibition Mechanism of RNA-Dependent RNA Polymerase (RdRp) of SARS-CoV-2 by Remdesivir and Nucleotide Analogues: A Molecular Dynamics Simulation Study. J. Phys. Chem. B.

[52] Gleeson M.P. (2008). Generation of a set of simple, interpretable ADMET rules of thumb. J. Med. Chem..

[53] Brandsma I., Derr R., Zhang G., Moelijker N., Hendriks G., Østerlund T. (2022). (Genotoxicity assessment of potentially mutagenic nucleoside analogues using ToxTracker^®^. Toxicol. Lett..

[54] Nabati M., Parsaee H. (2022). Potential cardiotoxic effects of remdesivir on cardiovascular system: A literature review. Cardiovasc. Toxicol..

[55] Hughes J.D., Blagg J., Price D.A., Bailey S., DeCrescenzo G.A., Devraj R.V., Ellsworth E., Fobian Y.M., Gibbs M.E., Gilles R.W. (2008). Physiochemical drug properties associated with in vivo toxicological outcomes. Bioorg. Med. Chem. Lett..

[56] Ertl P., Schuffenhauer A. (2009). Estimation of synthetic accessibility score of drug-like molecules based on molecular complexity and fragment contributions. J. Cheminform..

[57] Aleem A., Mahadevaiah G., Shariff N., Kothadia J.P. (2021). Hepatic manifestations of COVID-19 and effect of remdesivir on liver function in patients with COVID-19 illness. Baylor University Medical Center Proceedings.

[58] Griffiths S.K., Campbell J.P. (2015). Placental structure, function and drug transfer. Contin. Educ. Anaesth. Crit. Care Pain.

[59] Gao Y., Yan L., Huang Y., Liu F., Zhao Y., Cao L., Wang T., Sun Q., Ming Z., Zhang L. (2020). Structure of the RNA-dependent RNA polymerase from COVID-19 virus. Science.

[60] Xiong G., Wu Z., Yi J., Fu L., Yang Z., Hsieh C., Cao D. (2021). ADMETlab 2.0: An integrated online platform for accurate and comprehensive predictions of ADMET properties. Nucleic Acids Res..

[61] Kouranov A., Xie L., de la Cruz J., Chen L., Westbrook J., Bourne P.E., Berman H.M. (2006). The RCSB PDB information portal for structural genomics. Nucleic Acids Res..

[62] Kumar S.P., Patel C.N., Rawal R.M., Pandya H.A. (2020). Energetic contributions of amino acid residues and its cross-talk to delineate ligand-binding mechanism. Proteins Struct. Funct. Bioinform..

[63] Krieger E., Koraimann G., Vriend G. (2002). Increasing the precision of comparative models with YASARA NOVA—A self-parameterizing force field. Proteins Struct. Funct. Bioinform..

[64] Kerwin S.M. (2010). ChemBioOffice Ultra 2010 Suite.

[65] Tian W., Chen C., Liang J. (2018). CASTp 3.0: Computed atlas of surface topography of proteins and beyond. Biophys. J..

[66] Xu Y., Wang S., Hu Q., Gao S., Ma X., Zhang W., Shen Y., Chen F., Lai L., Pei J. (2018). CavityPlus: A web server for protein cavity detection with pharmacophore modelling, allosteric site identification and covalent ligand binding ability prediction. Nucleic Acids Res..

[67] Morris G., Goodsell D.S., Halliday R.S., Huey R., Hart W.E., Belew R.K., Olson A.J. (1998). Automated Docking Using a Lamarckian Genetic Algorithm and Empirical Binding Free Energy Function. J. Comput. Chem..

[68] DeLano W.L. (2002). Pymol: An open-source molecular graphics tool. CCP4 Newsl. Protein Crystallogr..

[69] Raya A., Barrientos-Salcedo C., Rubio-Póo C., Soriano-Correa C. (2011). Electronic structure evaluation through quantum chemical descriptors of 17β-aminoestrogens with an anticoagulant effect. Eur. J. Med. Chem..

[70] Frisch A. (2009). Gaussian 09W Reference.

[71] Dennington R., Keith T., Millam J. (2016). Gauss View 6.0.

[72] Humphrey W., Dalke A., Schulten K. (1996). VMD: Visual molecular dynamics. J. Mol. Graph..

[73] Lu T., Chen F. (2012). Multiwfn: A multifunctional wavefunction analyzer. J. Comput. Chem..

[74] Becke A.D. (1993). Density-functional thermochemistry. III. The role of exact exchange. J. Chem. Phys..

[75] Lee C., Yang W., Parr R.G. (1988). Development of the Colle-Salvetti correlation-energy formula into a functional of the electron density. Phys. Rev. B.

[76] Sundaraganesan N., Ilakiamani S., Dominic Joshua B. (2007). FT-Raman and FT-IR spectra, ab initio and density functional studies of 2-amino-4,5-difluorobenzoic acid. Spectrochim. Acta A Mol. Biomol. Spectrosc..

[77] Izadyar M., Khavani M., Housaindokht M.R. (2017). Sensing Ability of Hybrid Cyclic Nanopeptides Based on Thiourea Cryptands for Different Ions, A Joint DFT-D3/MD Study. J. Phys. Chem. A.

[78] Jasmine N.J., Muthiah P.T., Arunagiri C., Subashini A. (2015). Vibrational spectra (experimental and theoretical), molecular structure, natural bond orbital, HOMO-LUMO energy, Mulliken charge and thermodynamic analysis of N’-hydroxy-pyrimidine-2-carboximidamide by DFT approach. Spectrochim. Acta A Mol. Biomol. Spectrosc..

[79] Land H., Humble M.S. (2018). YASARA: A tool to obtain structural guidance in biocatalytic investigations. Protein Engineering: Methods and Protocols.

[80] Wang J., Wolf R.M., Caldwell J.W., Kollman P.A., Case D.A. (2004). Development and testing of a general amber force field. J. Comput. Chem..

[81] Essmann U., Perera L., Berkowitz M.L., Darden T., Lee H., Pedersen L.G. (1995). A smooth particle mesh Ewald method. J. Chem. Phys..

[82] Harrach M.F., Drossel B. (2014). Structure and dynamics of TIP3P, TIP4P, and TIP5P water near smooth and atomistic walls of different hydroaffinity. J. Chem. Phys..

[83] Tomarchio R., Patamia V., Zagni C., Crocetti L., Cilibrizzi A., Floresta G., Rescifina A. (2023). Steered Molecular Dynamics Simulations Study on FABP4 Inhibitors. Molecules.

[84] Lee J., Cheng X., Swails J.M., Yeom M.S., Eastman P.K., Lemkul J.A., Im W. (2016). CHARMM-GUI input generator for NAMD, GROMACS, AMBER, OpenMM, and CHARMM/OpenMM Simulations Using the CHARMM36 Additive Force Field. J. Chem. Theory Comput..

[85] Feller S.E., Zhang Y., Pastor R.W., Brooks B.R. (1995). Constant pressure molecular dynamics simulation: The Langevin piston method. J. Chem. Phys..

[86] Friesner R.A., Banks J.L., Murphy R.B., Halgren T.A., Klicic J.J., Mainz D.T., Shenkin P.S. (2004). Glide: A new approach for rapid, accurate docking and scoring. 1. Method and assessment of docking accuracy. J. Med. Chem..

[87] Hawkins G.D., Cramer C.J., Truhlar D.G. (1996). Parametrized models of aqueous free energies of solvation based on pairwise descreening of solute atomic charges from a dielectric medium. J. Phys. Chem..

[88] Genheden S., Kuhn O., Mikulskis P., Hoffmann D., Ryde U. (2012). The normal-mode entropy in the MM/PBSA method: Effect of system truncation, buffer region, and dielectric constant. J. Chem. Inf. Model..

[89] Pires D.E., Blundell T.L., Ascher D.B. (2015). pkCSM: Predicting small-molecule pharmacokinetic and toxicity properties using graph-based signatures. J. Med. Chem..

[90] Daina A., Michielin O., Zoete V. (2017). SwissADME: A free web tool to evaluate pharmacokinetics, drug-likeness and medicinal chemistry friendliness of small molecules. Sci. Rep..

[91] Lipinski C.A., Lombardo F., Dominy B.W., Feeney P.J. (1997). Experimental and computational approaches to estimate solubility and permeability in drug discovery and development settings. Adv. Drug Deliv. Rev..

