# Supplementary material for: De Novo Potent Peptide Nucleic Acid Antisense Oligomer Inhibitors Targeting SARS-CoV-2 RNA-Dependent RNA Polymerase via Structure-Guided Drug Design
Source: Int J Mol Sci. 2023 Dec 14;24(24):17473. doi: 10.3390/ijms242417473 (PMC10744289; doi:10.3390/ijms242417473)
Supplement: Supplementary file 1 [file ijms-24-17473-s001.zip › ijms-2696187-supplementary.pdf]

De Novo Potent Peptide Nucleic Acid Antisense Oligomer Inhibitors Targeting SARS-CoV-2 RNA-Dependent RNA Polymerase via Structure-Guided Drug Design

Kiran Shehzadi, Mingjia Yu * and Jianhua Liang *

Key Laboratory of Medical Molecule Science and Pharmaceutical Engineering, Ministry of Industry and Information Technology, School of Chemistry and Chemical Engineering, Beijing Institute of Technology, Beijing 10081, China; kiranshehzadi@bit.edu.cn
* Correspondence: 6120210204@bit.edu.cn (M.Y.); ljhbit@bit.edu.cn (J.L.)

Abstract: Global reports of novel SARS-CoV-2 variants and recurrence cases continue despite substantial vaccination campaigns, raising severe concerns about COVID-19. While repurposed drugs offer some treatment options for COVID-19, notably, nucleoside inhibitors like Remdesivir stand out as curative therapies for COVID-19 that are approved by the US Food and Drug Administration (FDA). The emergence of highly contagious SARS-CoV-2 variants underscores the imperative for antiviral drugs adaptable to evolving viral mutations. RNA-dependent RNA polymerase (RdRp) plays a key role in viral genome replication. Currently, inhibiting viral RdRp function remains a pivotal strategy to tackle the notorious virus. Peptide nucleic acid (PNA) therapy shows promise by effectively targeting specific genome regions, reducing viral replication, and inhibiting infection. In our study, we designed PNA antisense oligomers conjugated with cell-penetrating peptides (CPP) aiming to evaluate their antiviral effects against RdRp target using structure-guided drug design, which involves molecular docking simulations, drug likeliness and pharmacokinetic evaluations, molecular dynamics simulations, and computing binding free energy. The in silico analysis predicts that chemically modified PNAs might act as antisense molecules in order to disrupt ribosome assembly at RdRp's translation start site, and their chemically stable and neutral backbone might enhance sequence-specific RNA binding interaction. Notably, our findings demonstrate that PNA-peptide conjugates might be the most promising inhibitors of SARS-CoV-2 RdRp, with superior binding free energy compared to Remdesivir in the current COVID-19 medication. Specifically, PNA-CPP-1 could bind simultaneously to the active site residues of RdRp protein and sequence-specific RdRp-RNA target in order to control viral replication.

Keywords: peptide nucleic acid; cell-penetrating peptide; RdRp; RdRp-RNA; SARS-CoV-2

List of Figures:

Figure S1. Superposition of Cryo-EM structures of RdRp and RdRp-RNA……………………………………3

Figure S2. Targeted gene sequence of SARS-CoV-2 RdRp used for designed complimentary PNA analogues…………………………………………………………………………………………..4
Figure S3. The interaction analysis of PNA-CPP-1 with SARS-CoV-2 RdRp (blind docking analysis)..........5
Figure S4. The 2D diagram of interactions between PNA-CPP-1 and RdRp...6
Figure S5. The 2D diagram of interactions between PNA-CPP-1 lead compound and RdRp-RNA target...7
Figure S6. The interaction analysis of natural nucleotide and PNA-CPP-1 with SARS-CoV-2 RdRp-RNA…………………………………………………………………………………………………..8
Figure S7. The interaction analysis of ATP with RdRp-RNA and RdRp-RNA-PNA complex……………..8
Figure S8. Validation of same binding cavity by superimposition of Remdesivir and PNA-CPP-1 complex with RdRp-RNA (PDB ID: 7BV2).. 9
Figure S9. Validation of same binding cavity by superimposition of Remdesivir and PNA-CPP-1 complex with RdRp (PDB ID: 7BTF)..9
Figure S10. Cα distances of the binding pocket residues of PNA-CPP-1-RdRp-RNA (PDB ID: 7BV2) complex during SMD ((A) 6 pN, (B) 8 pN, (C) 10 pN) simulations...10
Figure S11. Cα distances of the binding pocket residues of PNA-CPP-1-RdRp (PDB ID: 7BTF) complex during SMD ((A) 6 pN, (B) 8 pN, (C) 10 pN) simulations...11
Figure S12. Radius of gyration (Rg) of PNA-CPP-1 with RdRp and RdRp-RNA..12
Figure S13. SASA analysis of PNA-CPP-1 complexes.. 12
Figure S14. The RMSD profiles of 800 ns MD simulations of unbound receptor (RdRp) and ligand (PNA-CPP-1)...13
Figure S15. Radius of gyration (Rg) of RdRp in the absence of PNA-CPP-1 during 800 ns MD simulation. .... **Error! Bookmark not defined.**
Figure S16. Interaction analysis of two complexes of PNA-CPP-1 with RdRp-RNA (PDB ID: 7BV2) including the initial and final structures obtained from MD…………………………………………………14
Figure S17. The interaction analysis of Remdesivir and PNA-CPP-1 with SARS-CoV-2 RdRp-RNA..........15
Figure S18. The 3D interaction analysis indicating distance between the atoms that form hydrogen bonds.. 16
Figure S19. The evolution of interatomic distances between chemical functions of the PNA-CPP-1 ligand and key amino acids residues in the binding sites during molecular dynamic simulation............................17
Figure S20. MMPBSA analysis of PNA-CPP-1-RdRp and PNA-CPP-1-RdRp-RNA complex………………18

List of Tables:

Table S1. The binding free energy PNA-CPP-1 with RdRp and RdRp-RNA by docking and MMPBSA analysis..18
Table S2. Potential drug binding cavities of RdRp detected by Cavity Plus. The amino acid residues of druggable cavities 1, 2, 3, and 4. 19
Table S3. The detailed information on non-bond forces of PNA-CPP-1 leading compound and SARS-CoV-2 RdRp in cavity 1 (PDB ID: 7BTF)..20

Table S4. The detailed information on non-bond forces of PNA-CPP-1 leading compound and SARS-CoV-2 RdRp in cavity 2 (PDB ID: 7BTF)..........21
Table S5. The detailed information on non-bond forces of PNA-CPP-1 leading compound and SARS-CoV-2 RdRp in cavity 3 (PDB ID: 7BTF)..........22
Table S6. The detailed information on non-bond forces of PNA-CPP-1 leading compound and SARS-CoV-2 RdRp in cavity 4 (PDB ID: 7BTF)..........24
Table S7. The detailed information on non-bond forces of PNA-CPP-1 leading compound in Remdesivir binding cavity targeting template RNA in complex with RdRp of SARS-CoV-2 (PDB ID: 7BV2)...............25

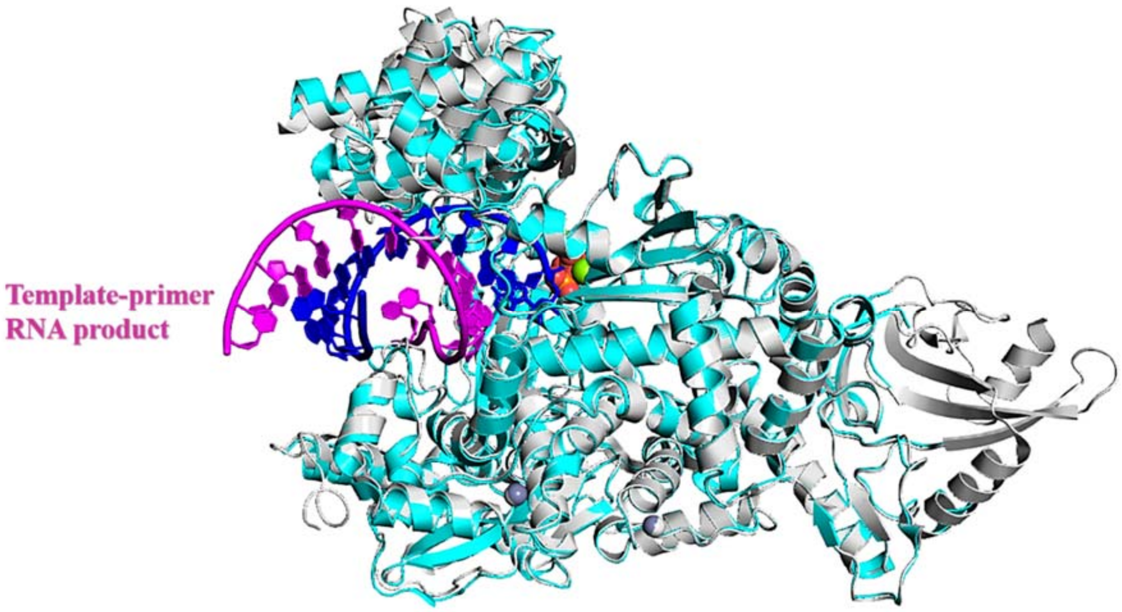

Figure S1. Superposition of Cryo-EM structures of RdRp and RdRp-RNA. RdRp (PDB ID: 7BTF, grey color) and RdRp bound to the template-primer RNA and triphosphate form of Remdesivir (PDB ID: 7BV2, cyan color). The RMSD value of systems aligned by PyMOL is 0.444.

Genome Sequence of SARS-CoV-2 RdRp: (NCBI Reference Sequence: NC_045512.2 from 13442 to 16236 bp)

TCAGCTGATGCACAATCGTTTTTAAACGGGTTTGCGGTGTAAGTGCAGCCCGTCTTACACCGTGCGGCACAGGCACTAGTACTG
ATGTCGTATACAGGGCTTTTGACATCTACAATGATAAAGTAGCTGGTTTTGCTAAATTCCTAAAAACTAATTGTTGTCGCTTCCAAG
AAAAGGACGAAGATGACAATTTAATTGATTCTTACTTTGTAGTTAAGAGACACACTTTCTCTAACTACCAACATGAAGAAACAATT
TATAATTTACTTAAGGATTGTCCAGCTGTTGCTAAACATGACTTCTTTAAGTTTAGAATAGACGGTGACATGGTACCACATATATCAC
GTCAACGTCTTACTAAATACACAATGGCAGACCTCGTCTATGCTTTAAGGCATTTTGATGAAGGTAATTGTGACACATTAAAAGAA
ATACTTGTCACATACAATTGTTGTGATGATGATTATTTCAATAAAAAGGACTGGTATGATTTTGTAGAAAACCCAGATATATTACGCG
TATACGCCAACTTAGGTGAACGTGTACGCCAAGCTTTGTTAAAAACAGTACAA

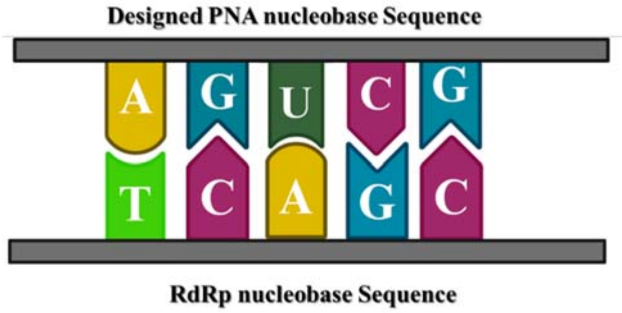

Figure S2. Targeted gene sequence of SARS-CoV-2 RdRp used for designed complimentary PNA analogues.

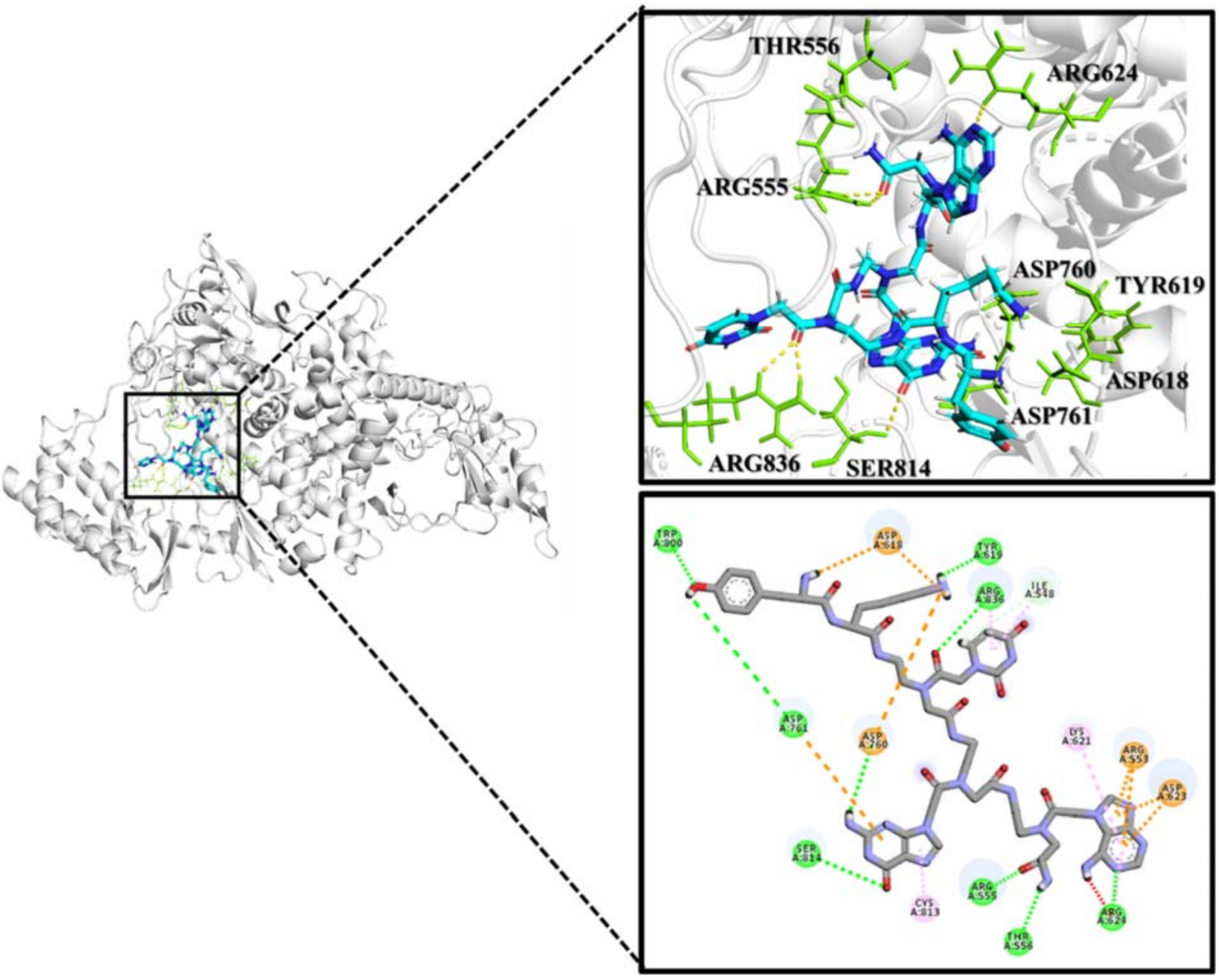

Figure S3. The interaction analysis of PNA-CPP-1 with SARS-CoV-2 RdRp (blind docking analysis). Close-up view of 3D and 2D interaction diagram of PNA-CPP-1 with RdRp (PDB ID: 7BTF). The Cartoon model represents the binding pocket of RdRp protein. PNA-CPP-1 bind at the experimental reported binding site of RdRp protein are represented as cartoon models. Amino acid form H-bond colored in green.

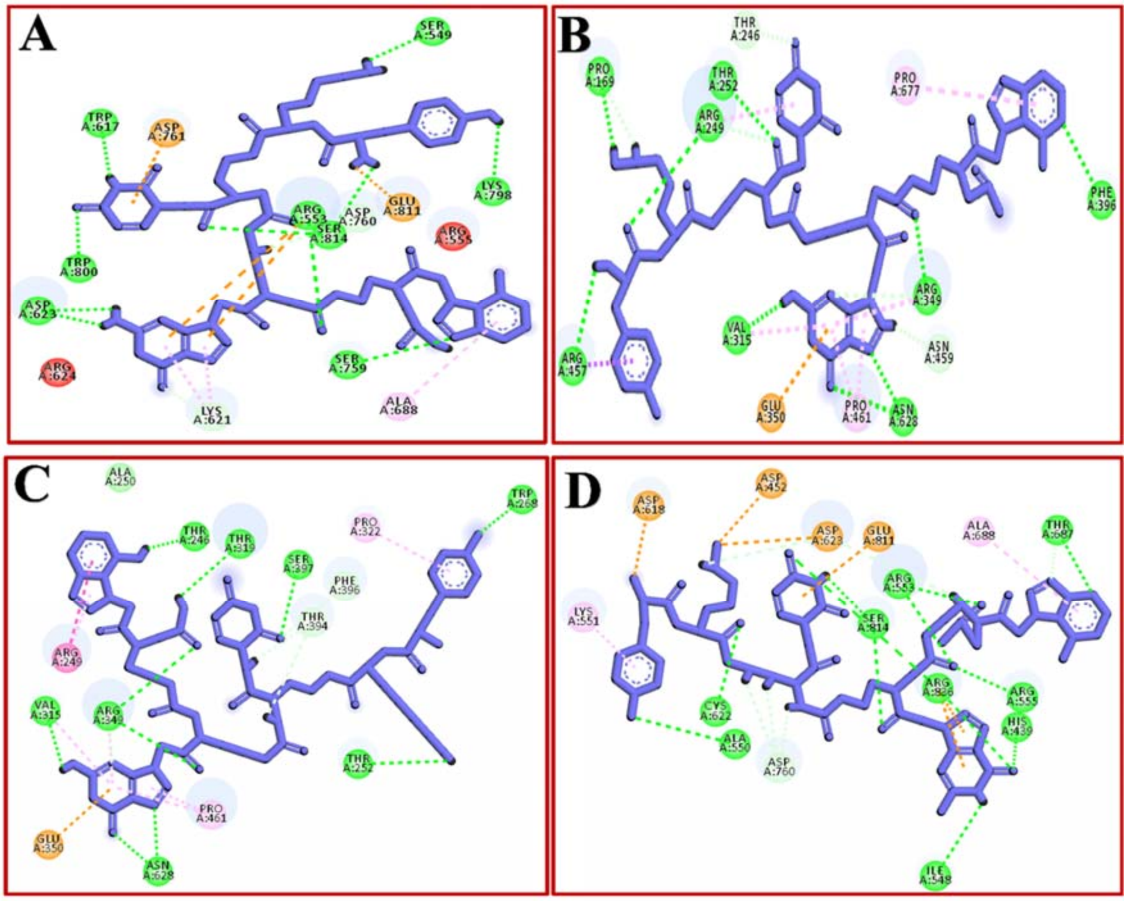

Figure S4. The 2D diagram of interactions between PNA-CPP-1 and RdRp. 2D interaction analysis of PNA-CPP-1(leading ligand) in the predicted binding cavities of RdRp (PDB ID: 7BTF) of SARS-CoV-2. **(A)** PNA-CPP-1 in druggable cavity1 of RdRp. **(B)** PNA-CPP-1 in druggable cavity 2 of RdRp. **(C)** PNA-CPP-1 in druggable cavity 3 of RdRp. **(D)** PNA-CPP-1 in druggable cavity 4 of RdRp. Green line showing conventional hydrogen bond with catalytic residues, orange and pink color represent the hydrophobic interactions.

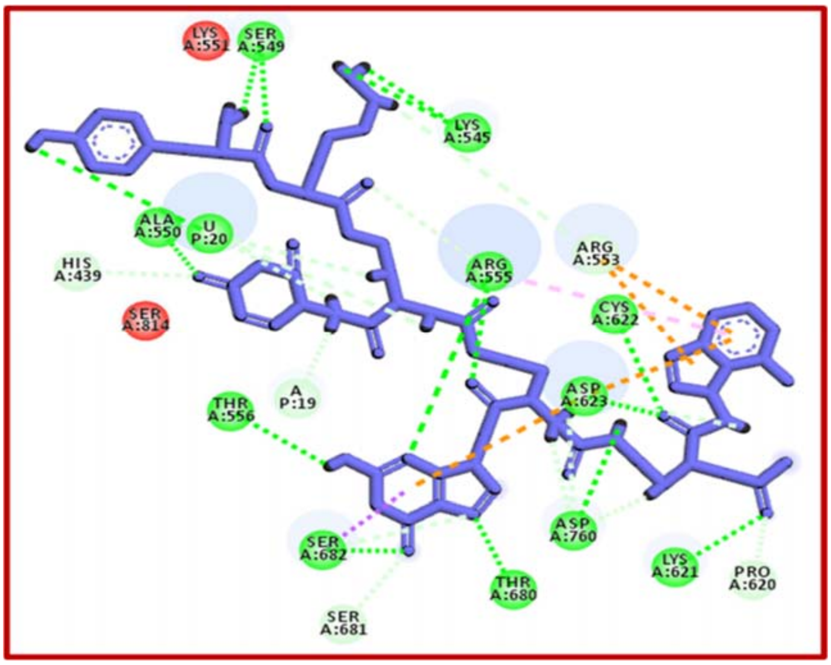

Figure S5. The 2D diagram of interactions between PNA-CPP-1 lead compound and RdRp-RNA target. 2D interaction analysis of PNA-CPP-1 binding at the growth site of RdRp-RNA.

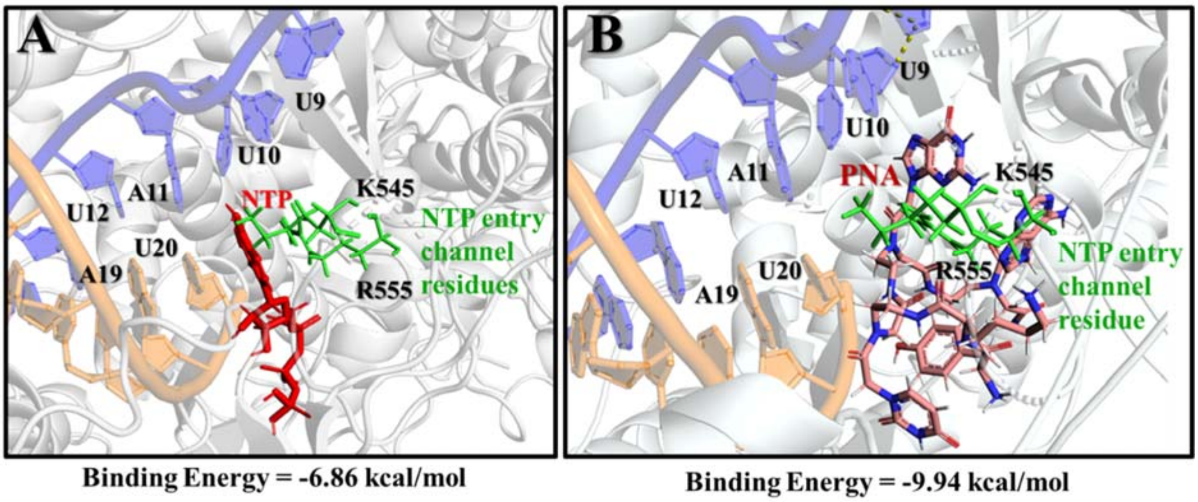

Figure S6. The interaction analysis of natural nucleotide and PNA-CPP-1 with SARS-CoV-2 RdRp-RNA. **(A)** Close-up view of 3D interaction diagram of ATP with RdRp-RNA (PDB ID: 7BV2) represents ATP incorporation into the primer strand of RNA and base-stacking interaction with U10 base of template strand. **(B)** Close-up view of 3D interaction diagram of PNA-CPP-1 with RdRp-RNA (PDB ID: 7BV2) represents PNA-CPP-1 incorporation into the primer strand of RNA at the place of ATP.

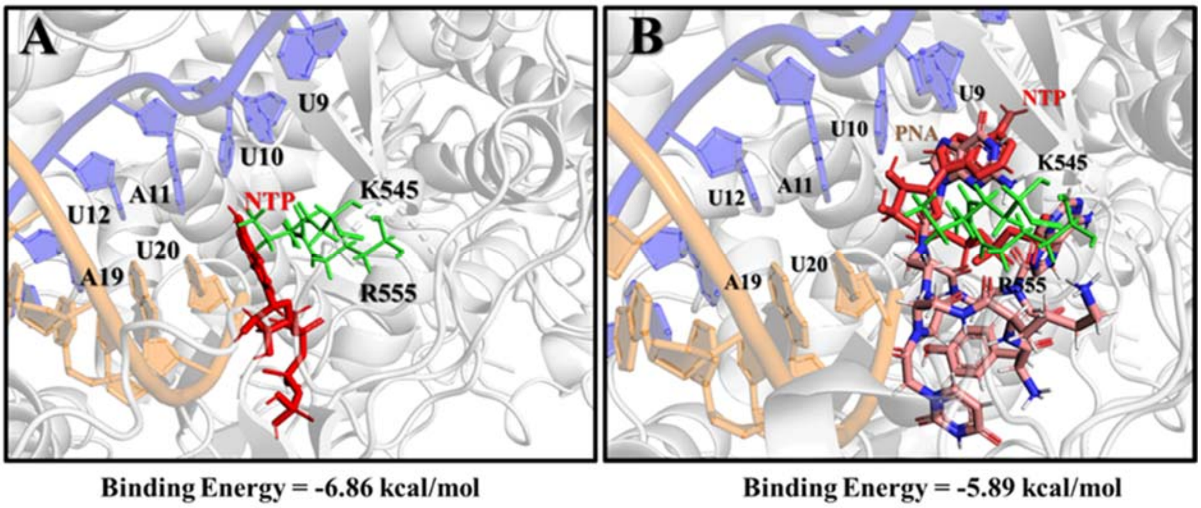

Figure S7. The interaction analysis of ATP with RdRp-RNA and RdRp-RNA-PNA complex. **(A)** Close-up view of 3D interaction diagram of ATP with RdRp-RNA (PDB ID: 7BV2) represents ATP incorporation into the primer strand of RNA **(B)** Close-up view of 3D interaction diagram of ATP with RdRp-RNA-PNA-CPP-1 complex represents PNA-CPP-1 incorporation into the primer strand of RNA at the place of ATP, upcoming ATP could not get incorporated at its proper place due to presence of PNA-CPP-1.

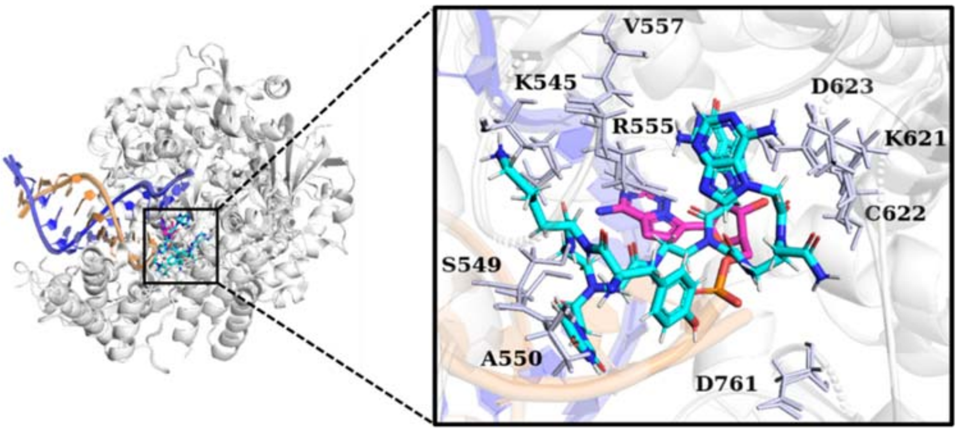

Figure S8. Validation of same binding cavity by superimposition of Remdesivir and PNA-CPP-1 complex with RdRp-RNA (PDB ID: 7BV2). PNA-CPP-1 inhibit SARS-CoV-2 transcription by interfering with the function of RNA on its growth site. The cartoon model represents the superimposition of the SARS-CoV-2 RdRp-RNA , shows the conservation of the Remdesivir (standard) binding site and PNA-CPP-1 (designed drug) binding site. Close-up view of the RdRp-RNA binding site, showing key amino acid residues (colored gray) interact with PNA-CPP-1 (colored in cyan) and Remdesivir (colored in purple). The RMSD value of systems aligned by PyMOL is 0.281.

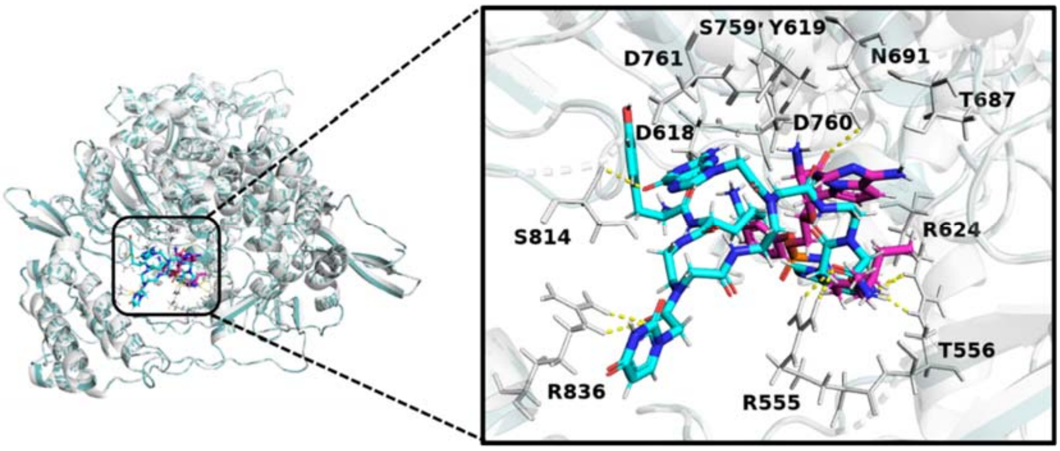

Figure S9. Validation of same binding cavity by superimposition of Remdesivir and PNA-CPP-1 complex with RdRp (PDB ID: 7BTF). The cartoon model represents the superimposition of the SARS-CoV-2 RdRp-RNA , shows the conservation of the Remdesivir (standard) binding site and PNA-CPP-1 (designed drug) binding site. Close-up view of the RdRp binding site, showing key amino acid residues (colored gray) interact with PNA-CPP-1 (colored in cyan) and Remdesivir (colored in purple). The RMSD value of systems aligned by PyMOL is 0.051.

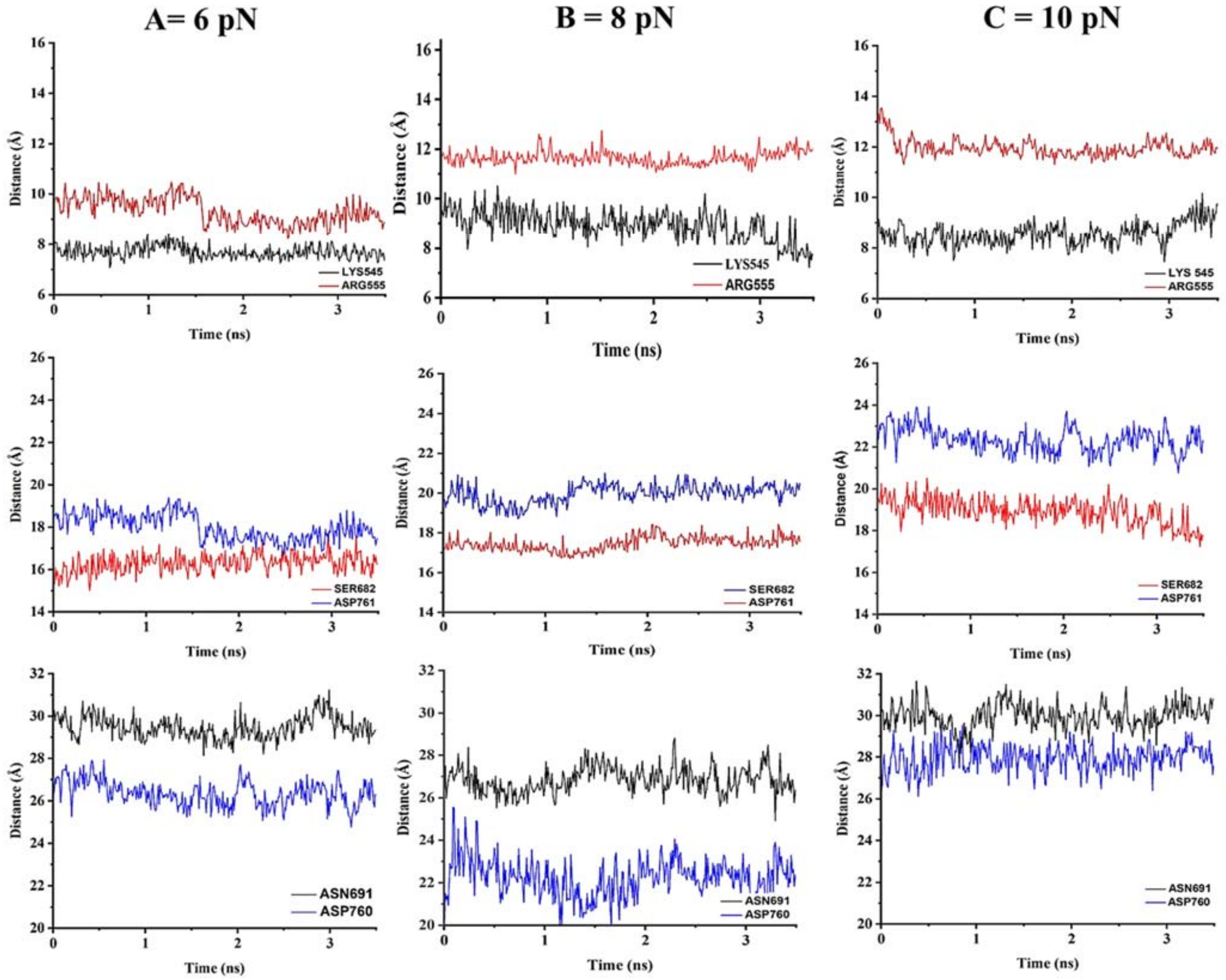

Figure S10. Cα distances of the binding pocket residues of PNA-CPP-1-RdRp-RNA (PDB ID: 7BV2) complex during SMD (**(A)** 6 pN, **(B)** 8 pN, **(C)** 10 pN) simulations.

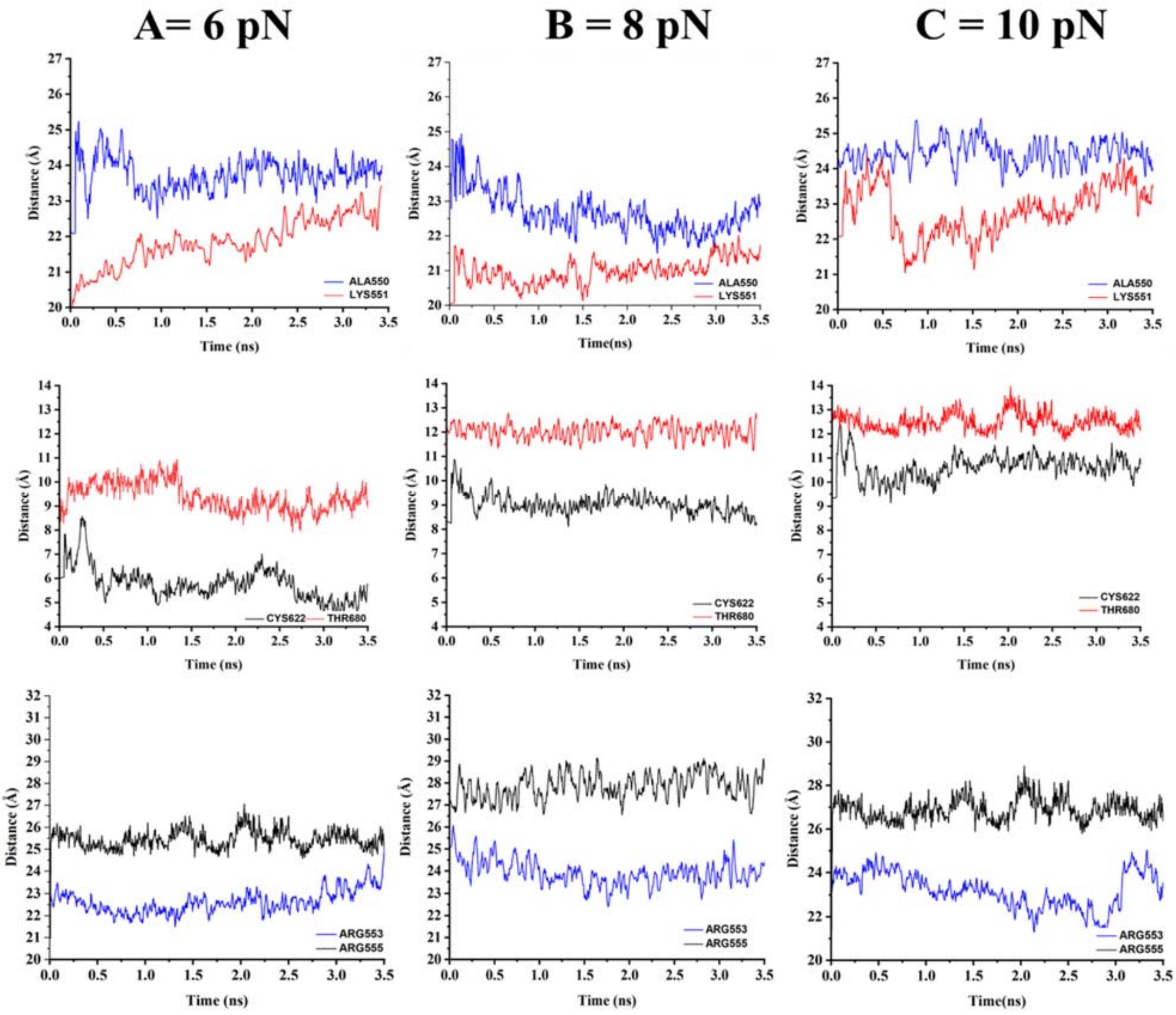

Figure S11. Cα distances of the binding pocket residues of PNA-CPP-1-RdRp (PDB ID: 7BTF) complex during SMD ((A) 6 pN, (B) 8 pN, (C) 10 pN) simulations.

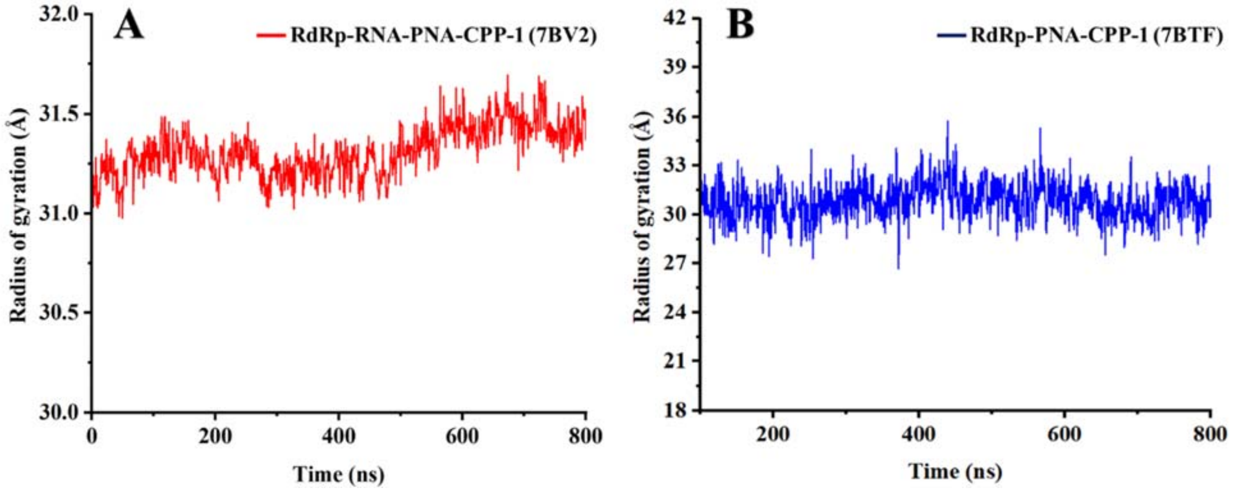

Figure S12. Radius of gyration (Rg) of PNA-CPP-1 with RdRp and RdRp-RNA. **(A)** Rg for RdRp-RNA with PNA-CPP-1 in red color. **(B)** Rg of RdRp-PNA-CPP-1 complex in blue color.

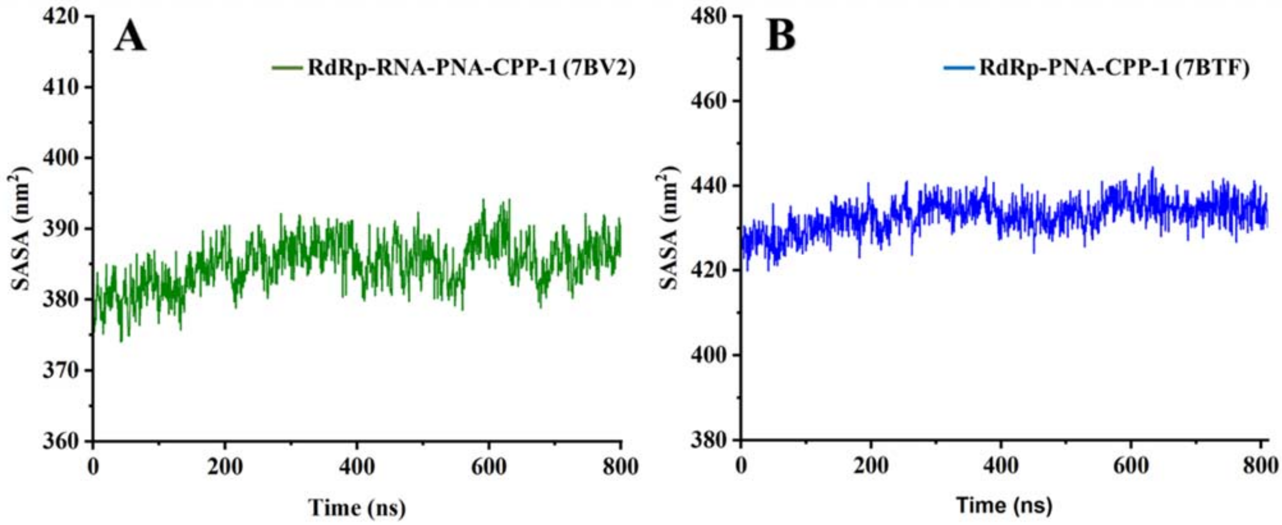

Figure S13. SASA analysis of PNA-CPP-1 complexes. The SASA curves highlighting the variation in accessibility of solvent for the complex with the receptor by means of MD simulation. **(A)** SASA of RdRp-RNA in complex with PNA-CPP-1 (PDB ID: 7BV2). **(B)** SASA OF RdRp in complex with PNA-CPP-1 (PDB ID: 7BTF).

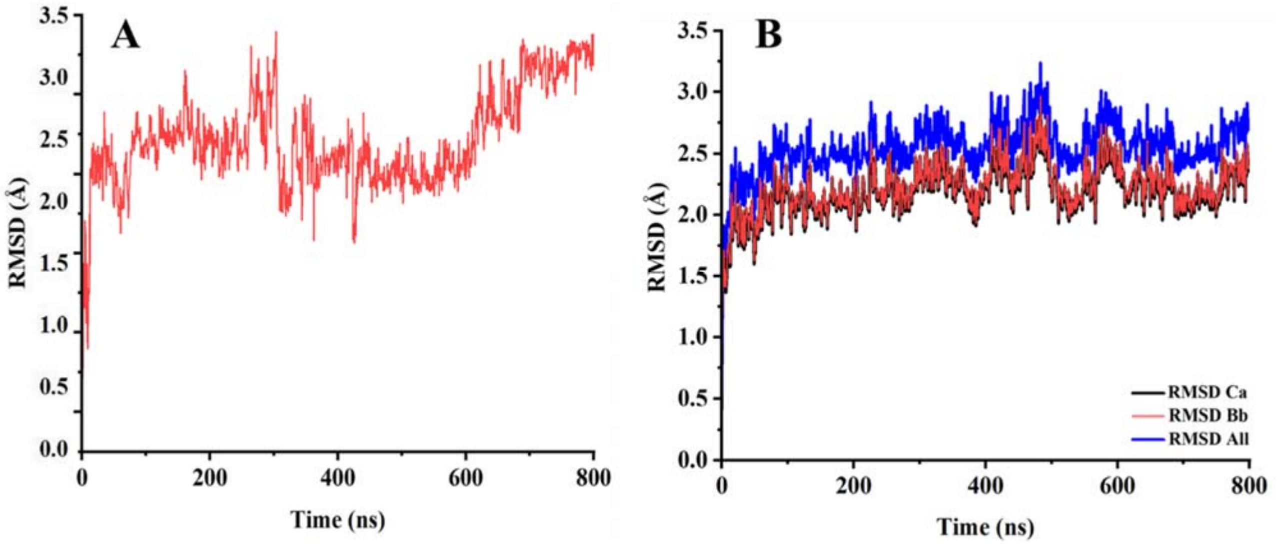

Figure S14. The RMSD profiles of 800 ns MD simulations of unbound receptor (RdRp) and ligand (PNA-CPP-1). **(A)** RMSD of all heavy atoms of ligand (colored in red). **(B)** RMSD of the C-Alpha of RdRp colored in black, backbone atoms colored in red and all heavy atoms colored in blue.

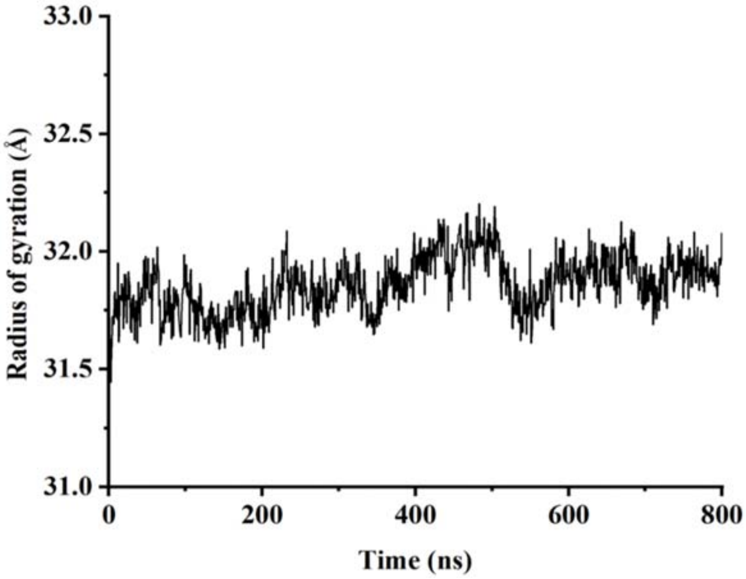

Figure S15. Radius of gyration (Rg) of RdRp in the absence of PNA-CPP-1 during 800 ns MD simulation.

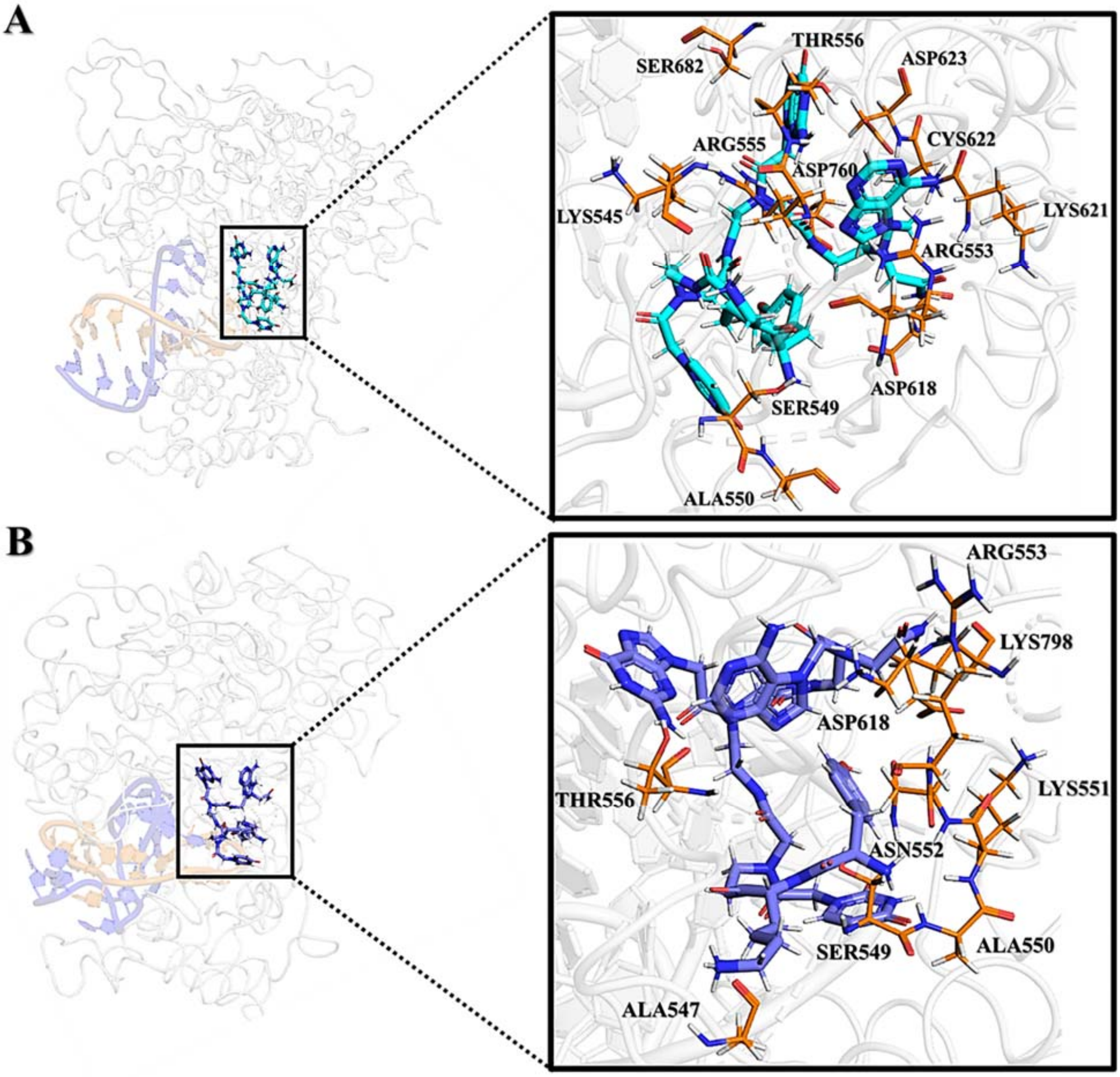

Figure S16. Interaction analysis of two complexes of PNA-CPP-1 with RdRp-RNA (PDB ID: 7BV2) including the initial and final structures obtained from MD. **(A)** PNA-CPP-1 complex and binding site residues (ligand colored in Cyan) at 0 ns. **(B)** PNA-CPP-1 complex and binding site residues (ligand colored in blue) at 800 ns. catalytic residue and other binding site residues are represented in orange sticks. Cartoon model at initial **(A)** and final **(B)** state of MD simulations depicted that PNA-CPP-1 remains in binding cavity with minor fluctuations.

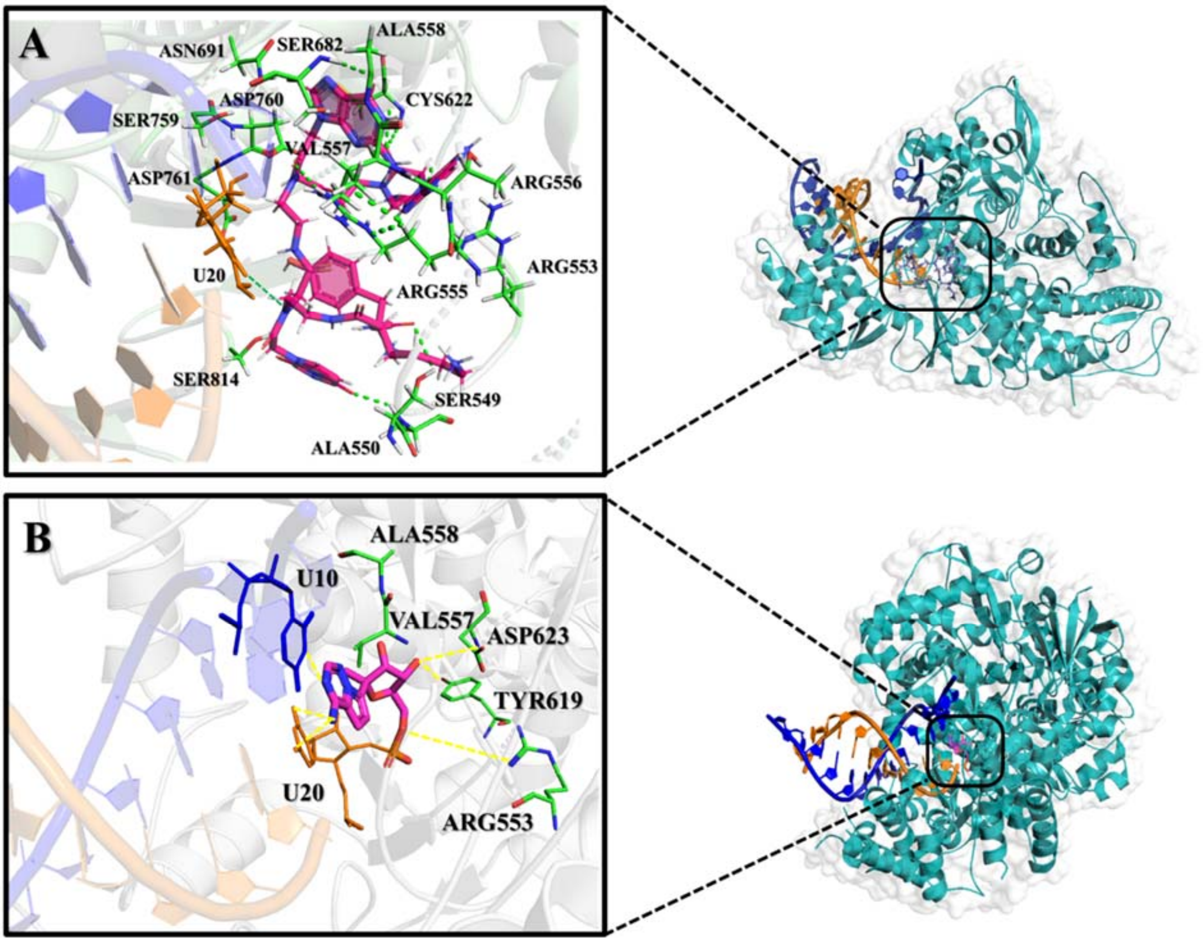

Figure S17. The interaction analysis of Remdesivir and PNA-CPP-1 with SARS-CoV-2 RdRp-RNA. **(A)** Close-up view of 3D model of the interaction between PNA-CPP-1 and RdRp-RNA (PDB ID: 7BV2). **(B)** Close-up view of 3D interaction diagram of Remdesivir with RdRp-RNA (PDB ID: 7BV2). The Cartoon model represents the catalytic part of RdRp-RNA. PNA-CPP-1 and Remdesivir bound at the same active sites of RdRp-RNA are represented as cartoon models. Amino acid and nucleotide untis which form H-bonds colored by yellow dotted lines.

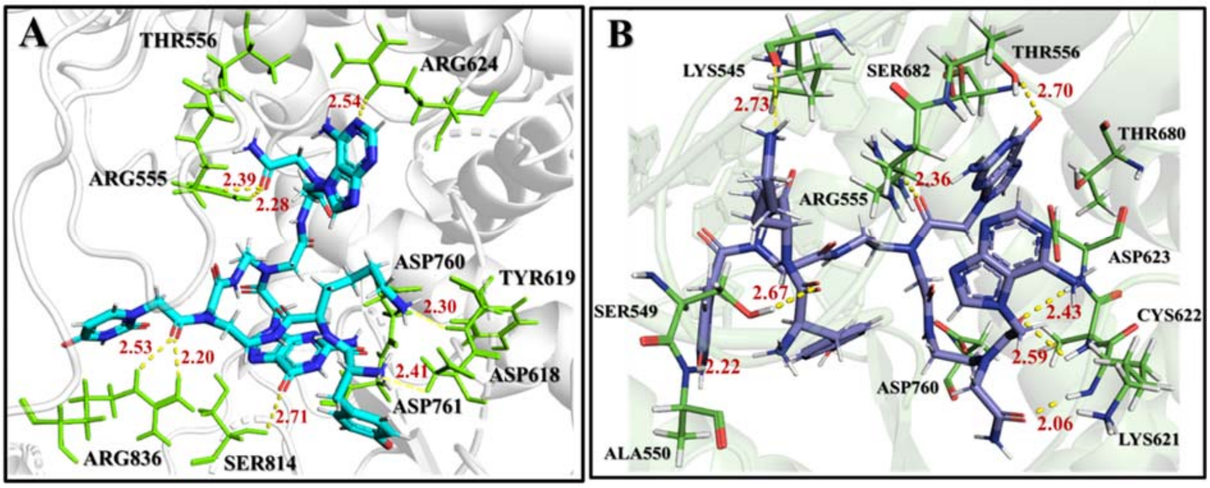

Figure S18. The 3D interaction analysis indicating distance between the atoms that form hydrogen bonds. **(A)** 3D Interaction analysis of PNA-CPP1 and the RdRp (PDB ID: 7BTF) showing interatomic distance between atoms that form H-bonds. **(B)** 3D Interaction analysis of PNA-CPP1 and the RdRp-RNA (PDB ID: 7BV2) showing interatomic distance between atoms that form H-bonds.

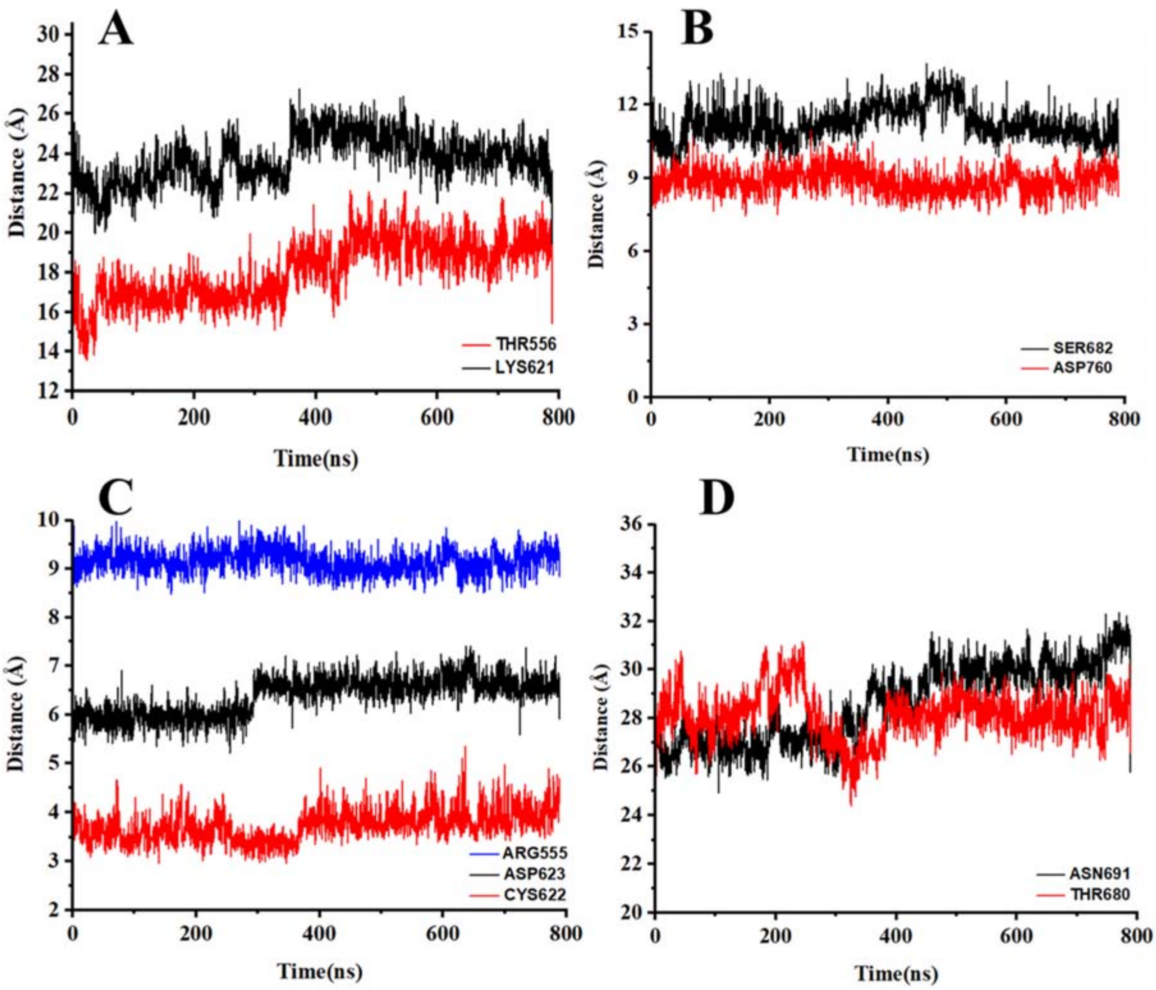

Figure S19. The evolution of interatomic distances between chemical functions of the PNA-CPP-1 ligand and key amino acids residues in the binding sites during molecular dynamic simulation. **(A)** LYS621 and THR556 represented the distance between O atom of PNA backbone and N atom of the pyrimidine ring respectively. **(B)** SER682 and ASP760 represented the distance between the O atom of carbonyl group attach with pyrimidine ring of guanine nucleobase and -NH group of PNA backbone respectively. **(C)** ARG555, ASP623, and CYS622 represented the distance between O atom of carbonyl group of PNA backbone, and guanine moiety. **(D)** ASN691, and THR680 represented the distance between N atom of the imidazole ring of nucleobases.

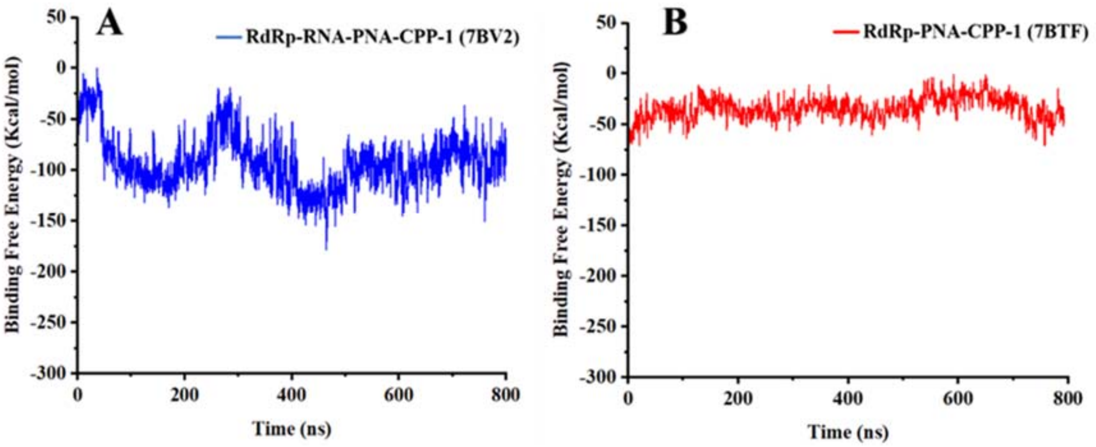

Figure S20. MMPBSA analysis of PNA-CPP-1-RdRp and PNA-CPP-1-RdRp-RNA complex. **(A)** Binding-free energy of top hit docked complex (PNA-CPP-1-RdRp-RNA) during 800 ns MD simulation colored in blue **(B)** Binding-free energy of top hit docked complex (PNA-CPP-1-RdRp) during 800 ns MD colored in red.

Table S1. The binding free energy PNA-CPP-1 with RdRp and RdRp-RNA by docking and MMPBSA analysis.

Target	Scoring (Kcal/mol)	PNA-CPP-1
RdRp	docking score (SP)	-9.40
	Binding energy (YASARA)	-9.19
	MMPBSA $\Delta G_{binding}$	-32.27
RdRp-RNA	docking score (SP)	-11.46
	Binding energy (YASARA)	-9.94
	MMPBSA $\Delta G_{binding}$	-92.06

Table S2. Potential drug binding cavities of RdRp detected by Cavity Plus. The amino acid residues of druggable cavities 1, 2, 3, and 4.

No. of Cavities	Amino acid residues of binding site
**Cavity 1**	ASP:161, TYR:163, ASP:164, PHE:165, VAL:166, GLU:167, GLN:408, THR:409, VAL:410, LYS:411, SER:434, LEU:437, LYS:438, HIS:439, PHE:441, GLN:444, SER:451, ASP:452, TYR:455, TYR:456, ARG:457, TYR:458, VAL:493, ILE:494, VAL:495, ASN:496, ASN:497, LEU:498, ASP:499, LYS:500, SER:501, ALA:502, GLY:503, PHE:506, ASN:507, LYS:508, TRP:509, GLY:510, LYS:511, ALA:512, TYR:516, GLN:541, MET:542, ASN:543, LEU:544, LYS:545, TYR:546, ALA:547, ILE:548, SER:549, ALA:550, LYS:551, ASN:552, ARG:553, ALA:554, ARG:555, THR:556, VAL:557, ALA:558, GLY:559, VAL:560, SER:561, ILE:562, SER:564, THR:565, MET:566, THR:567, ASN:568, ARG:569, GLN:570, PHE:571, HIS:572, GLN:573, LYS:574, LEU:575, LEU:576, LYS:577, SER:578, ILE:579, ALA:580, ALA:581, THR:582, ARG:583, VAL:588, ILE:589, GLY:590, THR:591, SER:592, LYS:593, PHE:594, TYR:595, GLY:597, TRP:598, MET:601, GLY:616, TRP:617, ASP:618, TYR:619, PRO:620, LYS:621, CYS:622, ASP:623, ARG:624, MET:626, GLU:665, LYS:676, GLY:679, THR:680, SER:681, SER:682, GLY:683, ASP:684, ALA:685, THR:686, THR:687, ALA:688, TYR:689, ASN:691, SER:692, MET:756, LEU:758, SER:759, ASP:760, ASP:761, ALA:762, VAL:763, VAL:792, PHE:793, MET:794, SER:795, GLU:796, ALA:797, LYS:798, CYS:799, TRP:800, THR:801, HIS:810, GLU:811, PHE:812, CYS:813, SER:814, GLN:815, HIS:816, PRO:830, TYR:831, PRO:832, ASP:833, PRO:834, SER:835, ARG:836, ILE:837, LEU:838, GLY:839, ALA:840, GLY:841, PHE:843, VAL:844, ASP:845, GLU:857, ARG:858, VAL:860, SER:861, LEU:862, ILE:864, ASP:865, ALA:866, TYR:877, PHE:920, MET:924, TYR:925, VAL:930, LEU:931, GLN:932.
**Cavity 2**	PHE:165, VAL:166, PRO:169, LEU:172, ARG:173, ALA:176, PRO:243, LEU:245, THR:246, LEU:247, THR:248, ARG:249, ALA:250, LEU:251, THR:252, ALA:253, GLU:254, SER:255, HIS:256, LEU:261, TYR:265, ILE:266, LYS:267, TRP:268, ASP:269, LEU:270, LEU:271, LYS:272, TYR:273, ASP:274, PHE:275, ALA:311, ASN:314, VAL:315, LEU:316, PHE:317, SER:318, THR:319, VAL:320, PHE:321, PRO:322, PRO:323, THR:324, SER:325, PHE:326, GLY:327, PRO:328, LEU:329, VAL:330, ARG:331, LYS:332, VAL:341, VAL:342, SER:343, THR:344, GLY:345, TYR:346, HIS:347, PHE:348, ARG:349, GLU:350, HIS:355, ASP:377, PRO:378, ALA:379, MET:380, HIS:381, ALA:382, ALA:383, SER:384, GLY:385, ASN:386, LEU:387, LEU:388, LEU:389, ASP:390, LYS:391, ARG:392, THR:393, THR:394, CYS:395, PHE:396, SER:397, VAL:398, ALA:399, ALA:400, TYR:453, ASP:454, TYR:455, TYR:456, ARG:457, TYR:458, ASN:459, LEU:460, PRO:461, THR:462, MET:463, CYS:464, PRO:537, MET:626, PRO:627, ASN:628, MET:629, LEU:630, CYS:659, ALA:660, LEU:663, SER:664, GLU:665, MET:666, LEU:673, TYR:674, VAL:675, LYS:676, PRO:677, GLY:678, ASN:791.
**Cavity 3**	PHE:165, VAL:166, ASN:168, PRO:169, ASP:170, LEU:172, ARG:173, ALA:176, ASN:177, GLY:179, PRO:243, ILE:244, LEU:245, THR:246, LEU:247, THR:248, ARG:249, ALA:250, LEU:251, THR:252, ALA:253, GLU:254, SER:255, HIS:256, LEU:261, PRO:264, TYR:265, ILE:266, LYS:267, TRP:268, LEU:270, LEU:271, LYS:272, TYR:273, ALA:311, ASN:314, VAL:315, LEU:316, PHE:317, SER:318, THR:319, VAL:320, PHE:321, PRO:322, PRO:323, THR:324, SER:325, PHE:326, GLY:327,

	PRO:328, LEU:329, VAL:330, VAL:341, SER:343, TYR:346, HIS:347, PHE:348, ARG:349, GLU:350, ASP:377, PRO:378, ALA:379, MET:380, HIS:381, ALA:382, ALA:383, LEU:387, LEU:388, LEU:389, ASP:390, LYS:391, ARG:392, THR:393, THR:394, CYS:395, PHE:396, SER:397, VAL:398, ALA:399, TYR:453, ASP:454, TYR:455, TYR:456, ARG:457, TYR:458, ASN:459, LEU:460, PRO:461, THR:462, MET:463, CYS:464, PRO:537, MET:626, PRO:627, ASN:628, MET:629, LEU:630, CYS:659, ALA:660, LEU:663, SER:664, GLU:665, MET:666, LEU:673, TYR:674, VAL:675, LYS:676, PRO:677, GLY:678, ASP:760, ASP:761, TYR:788, ASN:791.
**Cavity 4**	PHE:165, VAL:166, GLU:167, ASN:168, PRO:169, ASP:170, ILE:171, LEU:172, ARG:173, VAL:174, TYR:175, ALA:176, ASN:177, LEU:178, GLY:179, MET:242, PRO:243, ILE:244, LEU:245, THR:246, LEU:247, THR:248, ARG:249, ALA:250, LEU:251, THR:252, ALA:253, GLU:254, SER:255, HIS:256, LEU:261, TYR:265, ASN:314, VAL:315, LEU:316, PHE:317, SER:318, THR:319, VAL:320, PHE:321, PRO:322, PRO:323, THR:324, PHE:326, GLY:327, ARG:349, GLU:350, LYS:391, THR:393, THR:394, CYS:395, PHE:396, SER:397, TYR:453, ASP:454, TYR:455, TYR:456, ARG:457, TYR:458, ASN:459, LEU:460, PRO:461, THR:462, MET:463, CYS:464, LYS:545, TYR:546, ALA:547, ILE:548, SER:549, ALA:550, LYS:551, ASN:552, ARG:553, ALA:554, ARG:555, THR:556, VAL:557, ALA:558, GLY:559, CYS:622, MET:626, PRO:627, ASN:628, MET:629, LEU:663, SER:664, GLU:665, VAL:675, LYS:676, PRO:677, GLY:678, SER682, THR687, ASP:760, ASP:761, ALA:762, TYR:787, TYR:788, GLN:789, ASN:790, ASN:791.

Table S3. The detailed information on non-bond forces of PNA-CPP-1 leading compound and SARS-CoV-2 RdRp in cavity 1 (PDB ID: 7BTF).

Receptor [A]—Ligand [B]	Distance [Å]	Angle DHA [°]	Category
A: GLU811:OE2 - B: H493	1.98533	136.736	Salt Bridge; Attractive Charge
A: ARG553:HH21 - B: O16	2.08926	142.506	Conventional Hydrogen Bond
A: SER759: HG - B: N25	2.52932	124.675	Conventional Hydrogen Bond
A: TRP800:HE1 - B: O66	2.39831	153.698	Conventional Hydrogen Bond
A: SER814:HN - B: O59	3.07571	103.044	Conventional Hydrogen Bond
A: ASP623:OD1 - B: H411	2.95474	105.757	Conventional Hydrogen Bond
A: ASP623:OD2 - B: H412	2.64925	107.17	Conventional Hydrogen Bond
A: SER814: OG - B: H491	2.54071	90.249	Conventional Hydrogen Bond

A: TRP617: O - B: H63	1.99651	133.833	Conventional Hydrogen Bond
A: SER549: OG - B: H731	2.34924	129.922	Conventional Hydrogen Bond
A: LYS798: O - B: H81	2.25371	114.015	Conventional Hydrogen Bond
A: LYS62: HE2 - B: O40	2.81366	134.23	Carbon Hydrogen Bond
B: H122 - A: ASP760: OD2	3.00111	106.281	Carbon Hydrogen Bond
A: ARG553: NH1- B	4.21481	/	Pi-Cation
A: ARG553: NH1- B	3.58842	/	Pi-Cation
A: ASP761: OD2 - B	3.21058	/	Pi-Anion
A: LYS621- B	5.33223	/	Pi-Alkyl
A: ALA688 - B	5.12855	/	Pi-Alkyl
A: LYS621- B	5.28462	/	Pi-Alkyl

Table S4. The detailed information on non-bond forces of PNA-CPP-1 leading compound and SARS-CoV-2 RdRp in cavity 2 (PDB ID: 7BTF).

Receptor [A]—Ligand [B]	Distance [Å]	Angle DHA [°]	Category
A: ARG249: HH21- B: O74	3.00205	99.381	Conventional Hydrogen Bond
A: ARG249:HH22 - B: O74	2.95686	102.173	Conventional Hydrogen Bond
A: THR252:HG1 - B: O59	2.0322	158.612	Conventional Hydrogen Bond
A: ARG349:HH12 - B: O16	2.54329	108.585	Conventional Hydrogen Bond
A: ARG349:HH22 - B: O16	2.78656	106.967	Conventional Hydrogen Bond
A: PHE396:HN - B: N34	2.54927	125.013	Conventional Hydrogen Bond
A: ASN628:HD21- B: N30	2.52765	101.701	Conventional Hydrogen Bond
A: ASN628:HD22 - B: O40	2.53057	130.082	Conventional Hydrogen Bond
A: VAL315:O - B: H411	2.11805	162.513	Conventional Hydrogen Bond
A: ARG457:O - B: H491	2.81349	114.259	Conventional Hydrogen Bond

A: PRO169:O - B: H733	1.94454	156.735	Conventional Hydrogen Bond
A: THR246: HA - B: O66	2.60957	163.983	Carbon Hydrogen Bond
A: ARG249: HD2 - B: O59	2.25969	166.328	Carbon Hydrogen Bond
A: THR252: HB - B: O59	2.92931	114.47	Carbon Hydrogen Bond
A: ARG349: HD2 - B: N36	2.40314	128.731	Carbon Hydrogen Bond
A: PRO169:O - B: H733	1.94454	156.735	Conventional Hydrogen Bond
A: ASN459:OD1 - B: H31	3.04438	111.083	Carbon Hydrogen Bond
A: PRO169:O - B: H722	3.03134	110.864	Carbon Hydrogen Bond
A: GLU350:OE2 - B	4.66313	/	Pi-Anion
A: ARG457:HD1 - B	2.59809	/	Pi-Sigma
A: PRO461- B	4.15807	/	Pi-Alkyl
A: PRO677- B	5.48671	/	Pi-Alkyl
A: VAL315- B	5.25042	/	Pi-Alkyl
A: ARG349- B	4.48552	/	Pi-Alkyl
A: PRO461- B	3.87342	/	Pi-Alkyl
A: ARG249- B	3.56057	/	Pi-Alkyl
A: ARG457- B	4.26762	/	Pi-Alkyl

Table S5. The detailed information on non-bond forces of PNA-CPP-1 leading compound and SARS-CoV-2 RdRp in cavity 3 (PDB ID: 7BTF).

Receptor [A]—Ligand [B]	Distance [Å]	Angle DHA [°]	Category
A: TRP268:HN - B: O81	2.07776	152.111	Conventional Hydrogen Bond
A: ARG349:HH12 - B: O15	2.67535	116.35	Conventional Hydrogen Bond
A: ARG349:HH21 - B: O43	2.8454	90.709	Conventional Hydrogen Bond

A: SER397:HN - B: O65	2.26072	161.09	Conventional Hydrogen Bond
A: ASN628:HD21 - B: N30	2.7314	105.039	Conventional Hydrogen Bond
A: ASN628:HD22 - B: O40	2.35662	128.268	Conventional Hydrogen Bond
A: THR319:O - B: H11	2.69532	98.564	Conventional Hydrogen Bond
A: VAL315:O - B: H411	1.98844	151.848	Conventional Hydrogen Bond
A: THR246:O - B: H421	2.89504	103.669	Conventional Hydrogen Bond
A: THR252:O - B: H732	2.70463	135.433	Conventional Hydrogen Bond
A: THR252:OG1- B: H732	2.49399	123.768	Conventional Hydrogen Bond
A: ARG349:HD2 - B: N36	2.3887	132.252	Carbon Hydrogen Bond
A: PHE396: HA - B: O65	2.62652	153.446	Carbon Hydrogen Bond
A: THR394:O - B: H521	2.35297	166.252	Carbon Hydrogen Bond
A: THR394:O - B: H572	2.58983	145.601	Carbon Hydrogen Bond
A: GLU350:OE2 - B	4.66278	/	Pi-Anion
A: ARG249:C,O; ALA250: N - B	4.27009	/	Amide-Pi Stacked
A: ARG249- B	3.85385	/	Pi-Alkyl
A:PRO461- B	4.03475	/	Pi-Alkyl
A: ARG249 - B	3.73677	/	Pi-Alkyl
A: VAL315 - B	5.22027	/	Pi-Alkyl
A: ARG349 - B	4.44396	/	Pi-Alkyl
A:PRO461- B	3.898	/	Pi-Alkyl
A:PRO322 - B	4.97322	/	Pi-Alkyl

Table S6. The detailed information on non-bond forces of PNA-CPP-1 leading compound and SARS-CoV-2 RdRp in cavity 4 (PDB ID: 7BTF).

Receptor [A]—Ligand [B]	Distance [Å]	Angle DHA [°]	Category
A: ASP618:OD2 - B: N49	5.57538	/	Attractive Charge
A: ASP452:OD1 - B: N73	4.74484	/	Attractive Charge
A: ASP623:OD2 - B: N73	3.89186	/	Attractive Charge
A: HIS439:HD1 - B: O40	2.44846	134.671	Conventional Hydrogen Bond
A: ALA550:HN - B: O81	2.2957	126.482	Conventional Hydrogen Bond
A: ARG553:HH12 - B: O15	2.11746	145.774	Conventional Hydrogen Bond
A: ARG553:HH21 - B: O16	2.35137	133.684	Conventional Hydrogen Bond
A: ARG553:HH22 - B: O15	2.34747	138.296	Conventional Hydrogen Bond
A: ARG555:HH11 - B: O16	2.76977	97.714	Conventional Hydrogen Bond
A: CYS622:HN - B: O69	2.50517	166.842	Conventional Hydrogen Bond
A: THR687:HG1 - B: N32	2.1892	126.011	Conventional Hydrogen Bond
A: SER814: HG - B: O43	2.43739	100.341	Conventional Hydrogen Bond
A: ARG836: HE - B: O40	2.23134	163.437	Conventional Hydrogen Bond
A: ARG836:HH12 - B: O66	2.97092	109.798	Conventional Hydrogen Bond
A: ILE548:O - B: H38	2.82832	99.222	Conventional Hydrogen Bond
A: SER814: OG - B: H63	2.32227	144.661	Conventional Hydrogen Bond
A: THR687: HB - B: N25	2.9903	120.121	Carbon Hydrogen Bond
A: ASP623:OD2 - B: H51	2.99954	122.728	Carbon Hydrogen Bond
A: ASP760:O - B: H522	2.68174	136.194	Carbon Hydrogen Bond
A: ASP760:OD2 - B: H551	2.881	135.131	Carbon Hydrogen Bond
A: ASP760:OD2 - B: H671	2.98552	128.904	Carbon Hydrogen Bond

A: ASP623:OD2 - B: H721	2.86693	130.246	Carbon Hydrogen Bond
A: ARG836:NH1 - B	4.29272	/	Pi-Cation
A: ARG836:NH1 - B	4.47624	/	Pi-Cation
A: GLU811:OE2 - B	3.94431	/	Pi-Anion
A: ALA688 - B	5.15595	/	Pi-Alkyl
A: LYS551 - B	4.06753	/	Pi-Alkyl

Table S7. The detailed information on non-bond forces of PNA-CPP-1 leading compound in Remdesivir binding cavity targeting template RNA in complex with RdRp of SARS-CoV-2 (PDB ID: 7BV2).

Receptor [A]—Ligand [B]	Distance [Å]	Angle DHA [°]	Category
A: SER549: HG - B: O74	2.66643	99.709	Conventional Hydrogen Bond
A: ALA550:H - B: O66	2.21942	142.082	Conventional Hydrogen Bond
A: ARG555:HH21 - B: N36	2.76806	97.082	Conventional Hydrogen Bond
A: ARG555:HH22 - B: O43	2.26354	109.185	Conventional Hydrogen Bond
A: LYS621:H - B: O15	2.0563	164.407	Conventional Hydrogen Bond
A: CYS622:H - B: O21	2.59497	109.218	Conventional Hydrogen Bond
A:ASP623:H - B: O21	2.4264	161.396	Conventional Hydrogen Bond
A: THR680:HG1 - B: N30	2.56756	94.093	Conventional Hydrogen Bond
A: SER682:H - B: O40	2.36474	122.212	Conventional Hydrogen Bond
A: ASP760:OD2 - B:H7	2.33056	117.792	Conventional Hydrogen Bond
A: THR556:O - B:H411	2.78919	107.047	Conventional Hydrogen Bond
A: SER549: OG - B: H493	1.88017	146.29	Conventional Hydrogen Bond
A: LYS545:O - B: H732	2.73278	90.192	Conventional Hydrogen Bond
A: LYS545:O - B: H733	2.69832	92.118	Conventional Hydrogen Bond

P: U20:O1P - B: H81	2.21675	131.946	Conventional Hydrogen Bond
A: HIS439:HD2 - B: O66	2.95477	119.163	Carbon Hydrogen Bond
A: ARG555:HD2 - B: O69	2.32149	151.379	Carbon Hydrogen Bond
A:PRO620: HA - B: O15	2.3486	150.497	Carbon Hydrogen Bond
A: SER681: HA - B: O40	2.87001	128.163	Carbon Hydrogen Bond
A: SER682: HA - B: N30	2.96767	113.652	Carbon Hydrogen Bond
A: ASP760:OD2- B: H52	2.94577	111.914	Carbon Hydrogen Bond
A: ASP760:OD1- B: H91	2.8626	104.68	Carbon Hydrogen Bond
A: ASP761:OD1- B: H92	2.82419	107.19	Carbon Hydrogen Bond
A: ASP623:OD1- B:H192	3.09782	113.932	Carbon Hydrogen Bond
P: U20:O2P - B:H521	2.56451	164.966	Carbon Hydrogen Bond
P: U20:O4 - B:H551	2.62076	136.592	Carbon Hydrogen Bond
P: A19:O2P - B:H571	2.18767	173.207	Carbon Hydrogen Bond
P: U20:O2P - B:H572	2.54793	168.094	Carbon Hydrogen Bond
A: ARG553:O - B:H722	3.07145	121.398	Carbon Hydrogen Bond
A: ARG553:NH1 - B	3.38997	/	Pi-Cation
A: ARG553:NH2 - B	3.25016	/	Pi-Cation; Pi-Donor Hydrogen Bond
A: ASP623:OD1 - B	3.4964	/	Pi-Anion
A: ASP623:OD2 - B	3.20976	/	Pi-Anion
A: SER682:HB2 - B	2.7343	/	Pi-Sigma
A: ARG555 - B	5.46369	/	Pi-Alkyl
